# Structure–Function Relationships in the Human P-Glycoprotein (ABCB1): Insights from Molecular Dynamics Simulations

**DOI:** 10.3390/ijms23010362

**Published:** 2021-12-29

**Authors:** Liadys Mora Lagares, Yunierkis Pérez-Castillo, Nikola Minovski, Marjana Novič

**Affiliations:** 1 Theory Department, Laboratory for Cheminformatics, National Institute of Chemistry, 1000 Ljubljana, Slovenia; nikola.minovski@ki.si; 2 Jožef Stefan International Postgraduate School, 1000 Ljubljana, Slovenia; 3 Bio-Cheminformatics Research Group and Escuela de Ciencias Físicas y Matemáticas, Universidad de Las Américas, Quito 170513, Ecuador; yunierkis@gmail.com

**Keywords:** P-Glycoprotein, molecular dynamics simulations, ABC transporter, multidrug resistance (MDR), conformational changes

## Abstract

P-Glycoprotein (P-gp) is a transmembrane protein belonging to the ATP binding cassette superfamily of transporters, and it is a xenobiotic efflux pump that limits intracellular drug accumulation by pumping compounds out of cells. P-gp contributes to a reduction in toxicity, and has broad substrate specificity. It is involved in the failure of many cancer and antiviral chemotherapies due to the phenomenon of multidrug resistance (MDR), in which the membrane transporter removes chemotherapeutic drugs from target cells. Understanding the details of the ligand–P-gp interaction is therefore critical for the development of drugs that can overcome the MDR phenomenon, for the early identification of P-gp substrates that will help us to obtain a more effective prediction of toxicity, and for the subsequent outdesign of substrate properties if needed. In this work, a series of molecular dynamics (MD) simulations of human P-gp (*h*P-gp) in an explicit membrane-and-water environment were performed to investigate the effects of binding different compounds on the conformational dynamics of P-gp. The results revealed significant differences in the behaviour of P-gp in the presence of active and non-active compounds within the binding pocket, as different patterns of movement were identified that could be correlated with conformational changes leading to the activation of the translocation mechanism. The predicted ligand–P-gp interactions are in good agreement with the available experimental data, as well as the estimation of the binding-free energies of the studied complexes, demonstrating the validity of the results derived from the MD simulations.

## 1. Introduction

P-Glycoprotein (P-gp) is one of the most-studied membrane transporters that belong to the ATP-binding cassette (ABC) superfamily, probably because of the role it plays in multidrug resistance (MDR), a phenomenon in which there is cellular resistance to a variety of structurally and functionally unrelated chemotherapeutic agents. The identification of P-gp more than three decades ago enabled the discovery of the fact that the reduced intracellular accumulation of anticancer drugs can lead to significant levels of drug resistance [1,2]. Over the years, P-gp has become the prototype MDR transporter, with studies concluding that drug resistance associated with P-gp leads to major failures of chemotherapy in human cancers [3].

**Figure 1:**
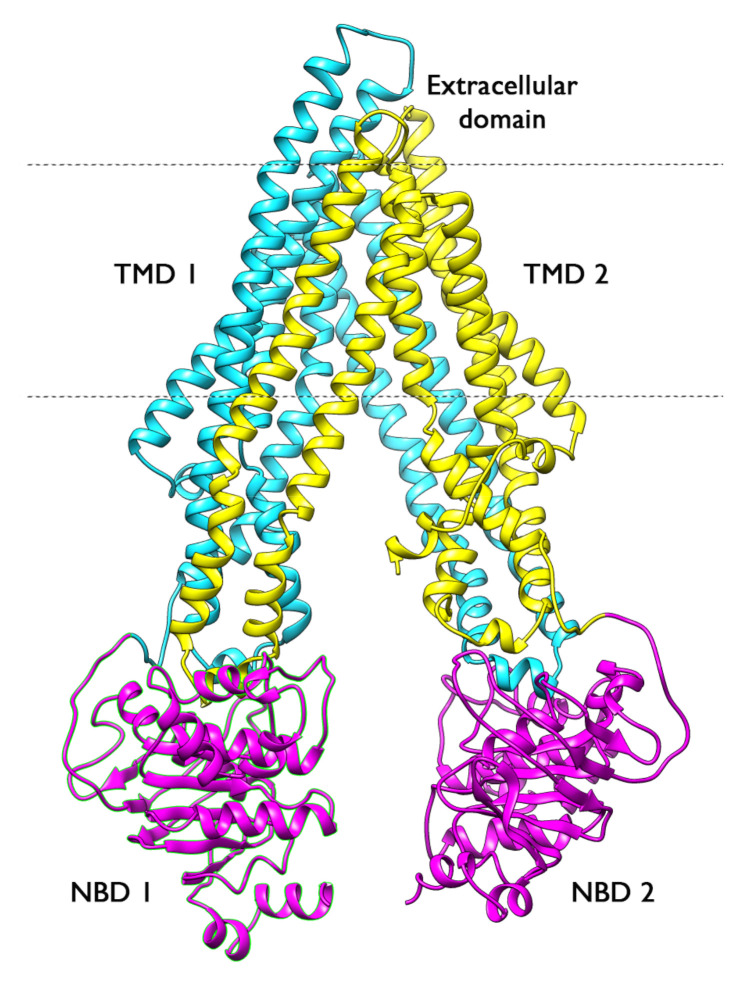
Crystal structure of mP-gp (PDB ID: 4M1M) in the inward-facing conformation. The NBDs are shown in magenta and the TMDs are shown in cyan and yellow.

P-gp is a 1280-residue single polypeptide with a molecular weight of 170 kDa. It contains two transmembrane domains (TMDs) and two cytosolic ATP-binding regions called nucleotide binding domains (NBDs) (Figure 1). These two symmetrical halves are connected by a highly charged “linker region” ∼75 amino acids in length [4]. Each TMD consists of six highly hydrophobic α-helices embedded in the membrane bilayer and extending into the cytosol to form the intracellular loops (ICLs). The TMDs form the pathway by which drug molecules cross the membrane, switching between inward- and outward-facing conformations [5]; although they are structurally similar across the transporter family, they have a large proportion of non-conserved amino acids. In contrast, the NBDs contain three highly conserved sequence motifs at which ATP is hydrolysed: the Walker A and Walker B motifs, and the ABC signature motif. The actual nucleotide binding site is shared by the NBDs and therefore arises only when they dimerize, consisting of the Walker A motif of one NBD and the ABC motif of the other NBD.

Although it has often been assumed that ATP hydrolysis drives the transport process, the major conformational changes leading to substrate transport occur after ATP binding, and not after ATP hydrolysis. Similarly, the reduction in the binding affinity of drugs to P-gp seems to be due to the ATP binding, which induces conformational changes exposing the binding site to the extracellular medium, rather than to hydrolysis [6,7,8]. ATP hydrolysis may be responsible for the resetting of the transporter to the initial conformation [9], allowing a new catalytic cycle to occur.

The transport cycle of P-gp can be stimulated or modulated by compounds that act as substrates or inhibitors [10], but the molecular mechanisms of the way in which this occurs are not fully understood. From this perspective, structural and dynamic insights into P-gp transport are essential to better understand how this transporter functions and, in this way, to facilitate the development of inhibitors which are relevant to the clinical practice that could overcome MDR, or the identification of P-gp ligands that could contribute to the more effective prediction of toxicity at early stages of drug development.

The molecular understanding of the transport mechanism of P-gp depends on the available experimental structural information that provides snapshots of the conformational cycle related to substrate transport. The diversity of conformations in which the P-gp structure has been solved demonstrates the flexibility of the transporter [11,12], a property associated with its polyspecificity, i.e., the ability to bind a large number of chemically diverse compounds. Over time, numerous ligand-based models have been developed to predict the P-gp activity, such as quantitative structure–activity relationship (QSAR) models [13,14,15,16] and structure-based models based on molecular docking and the use of the mouse P-gp (*m*P-gp) 3D structure [17,18], or homology models of the human P-gp (*h*P-gp) [19], in which the binding modes of substrates and inhibitors have been well characterized. Nonetheless, the molecular details of the effects of these molecules on the conformational dynamics of P-gp are not well known. Therefore, methods to study the dynamics at the atomistic level are needed in order to elucidate the conformational changes that P-gp undergoes during the translocation mechanism.

Under this framework, MD simulations have proven useful in biological and chemical studies because they can provide structural and dynamical information at the atomistic level. MD simulations have already been used to study P-gp drug interactions and other dynamic processes of P-gp [20]. Some studies have highlighted the potential of P-gp to accommodate multiple drug molecules simultaneously in the binding pocket, and others have described the importance of a lipid bilayer environment in the study of P-gp [21,22], revealing important details for the study of this transporter. However, as the human crystal structure of P-gp was not available at that time, the reported studies were performed either on homology models or on the crystal structure of mouse P-gp. Recently, in 2019, the cryo-electron microscopy (cryoEM) structure of *h*P-gp in the inward facing conformation was solved (PDB ID: 6QEX) [11], which raised much hope for better and more efficient development in the study of P-gp.

Here, we describe a series of MD simulations based on the human cryo-EM structure of P-gp, aimed at the analysis of the behaviour of different molecules (substrates, inhibitors, and non-active compounds) within the binding pocket, and evaluating their effects on the dynamics and conformations of NBDs and TMDs at the atomistic level. Another goal of the study was to identify the patterns of movement exhibited by the transporter in the context of the translocation process, which will allow the identification of the initial steps of the efflux mechanism.

## 2. Results and Discussion

Molecular dynamics simulations were performed on a series of ligand–P-gp complexes formed by nine different compounds (see Supplementary Materials Table S1), including four drugs known to interact with P-gp as substrates, inhibitors, or both: cyclosporin A (CSA), a high-affinity substrate [23] and inhibitor [24] of P-gp; amiodarone (AMI) [25], doxorubicin (DOX) [26,27], and carvedilol (CAR) [25], which are well-known substrates of P-gp; and five compounds that do not interact with P-gp: pamidronate (APD), busulfan (BUS), gentamicin (GEN), paraquat (PQT) [28], and valproic acid (VPA) [29,30,31]. We used the available knowledge on the interaction of substrates and inhibitors with P-gp to obtain a detailed description of the dynamics of the *h*P-gp at the atomistic level, which was previously limited to studies based on the structure of *m*P-gp or homology models of *h*P-gp.

### 2.1. Overall Systems Dynamics

All of the MD simulations were performed on systems that had reached a state of energetic equilibrium (see Supplementary Materials Figure S1).

**Figure 2:**
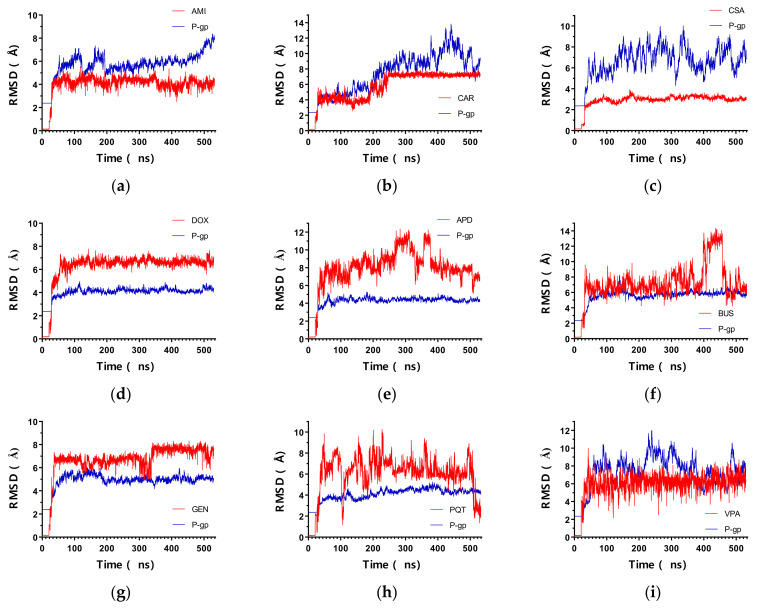
RMSD vs time of the simulated P-gp–ligand complexes: backbone atoms of the protein chains (blue) and ligand (red). (**a**) P-gp–AMI; (**b**) P-gp–CAR; (**c**) P-gp–CSA; (**d**) P-gp–DOX; (**e**) P-gp–APD; (**f**) P-gp–BUS; (**g**) P-gp–GEN; (**h**) P-gp–PQT; (**i**) P-gp–VPA.

Figure 2 shows the backbone RMSD for all of the systems during the 530 ns time of the simulation. As can be seen, the protein backbone RMSD showed a rapid increase in the first 30 ns, followed by a stable fluctuation throughout the course of all of the simulations, indicating that the conformational equilibrium was reached. The trajectories were stable during the 500 ns production run, fluctuating within a range of 3 Å after the initial equilibration phases for most of the complexes. As for the ligands, they remained docked at their respective binding sites during the simulation time, resulting in very low RMSD values. For the P-gp active ligands, their RMSD values were indicative of the stability of the complexes, as the fluctuations were minimal and ranged within 1 Å, whereas the RMSD for the non-active compounds showed larger fluctuations throughout the entire simulation. In general, the low positional deviations of the active ligands suggest a stable binding. Interestingly, for CAR, there was an increase in the RMSD values at about 180–250 ns, which then stabilized again until the end of the simulation. Looking at the trajectory, this coincided with a change in the conformation of CAR within the binding pocket: the molecule rotated such that the carbazole moiety interacted with residue Q990, and the methoxyphenyl ring interacted with residue I340. This conformation was maintained until the end of the simulation. Previously, residue Q990 interacted with the methoxyphenyl ring of CAR, and its carbazole moiety interacted with I340.

**Figure 3:**
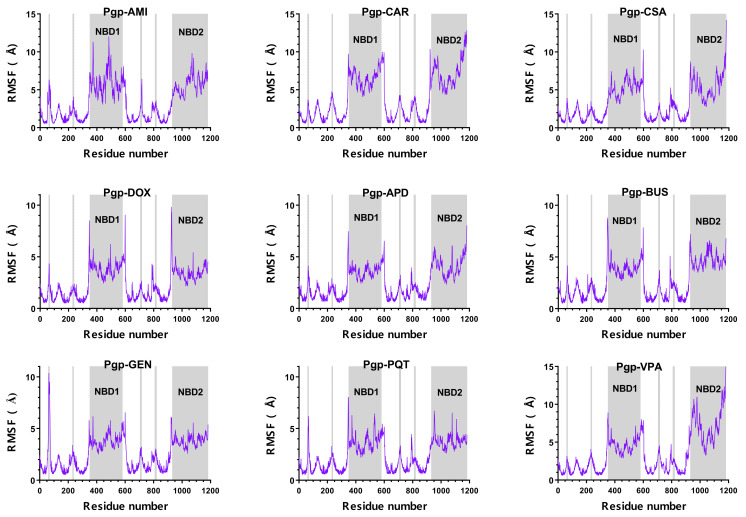
Per-residue RMSF for the simulated ligand–P-gp complexes. The residue numbers do not correspond to the IDs in the PDB file of the cryo-EM structure, but are consecutive, as required by the simulation software.

The high stability of the simulated complexes is further illustrated by the root-mean-square fluctuation (RMSF) plots (Figure 3), which show the fluctuations of each individual atom around its average position. The behaviour of the RMSF was similar for all of the systems; most of the residues of the protein fluctuated less than 3 Å relative to the average structure, and the RMSF peaks were associated with the same specific regions in all of the complexes studied. The loop regions and the NBDs exhibited the largest fluctuations, whereas the helices of the transmembrane domain remained stable throughout the simulation. In addition to the NBDs region, higher values of atomic fluctuations were also observed at one of the extracellular loops (around residues 90–100), which is important for the transport mechanism, as it allows rapid closure of the outward-facing conformation, preventing the re-entry of the substrate into the translocation pathway [12]. Smaller peaks were also observed for some of the intracellular loops between different transmembrane (TM) regions, e.g., TM4-TM5, TM8-TM9, TM10-TM11, and the helix breakers of TM4 and TM10. In the inward-facing conformation, TM4 and TM10 were interrupted by flexible loops that were thought to act as flexible hinges to open the drug-translocation pathway. Therefore, the observed coordinate fluctuations were in good agreement with the available literature [32,33]. 

**Figure 4:**
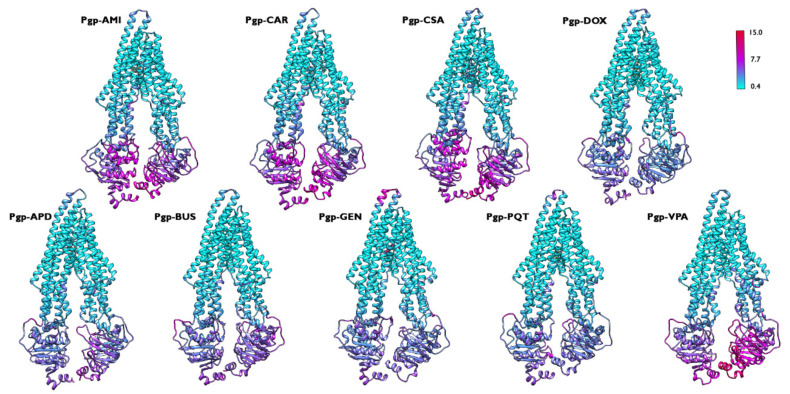
Backbone RMSF coloured representation for the simulated P-gp–ligand systems along the 500 ns production run. The flexibility scale goes from cyan (lower values) to red (higher values). The same regions are among the most flexible in all of the studied systems; however, the flexibility is higher for the active complexes.

Figure 4 provides an overview of the flexibility of the protein backbone in each simulated complex. Although the behaviour of the RMSF of the protein backbone was similar in all of the systems, there were some differences in the magnitude of the flexibility of the NBDs. The greater movement of both NBDs was expected for the activation of the translocation pathway, and the complexes formed by P-gp and the active compounds showed the higher flexibility of these domains compared to the non-active bound complexes. However, the system formed by P-gp and VPA displayed a greater flexibility for only one of the NBDs. The observed variation in the behaviour of this inactive complex, which may seem contradictory, could find an explanation in the properties of VPA, which has been described in some studies as an inducer of P-gp expression and function [30,31]. Therefore, its inductive nature could lead to the observed differences in the movement patterns, which were not fully consistent with the observations for purely active or inactive compounds.

### 2.2. Ligand–Protein Interactions

Only the interactions observed in more than 50% of the production snapshots were considered in the analysis. The observed ligand–P-gp interactions mainly involved residues in TM1, TM4, TM5, TM6, TM12, and to a lesser extent in TM3, TM7, TM10, and TM11 (see Supplementary Materials, Figure S2), which is consistent with our recent docking study showing the interactions with the same TMDs [19]. Some regions that interact exclusively with the active compounds have also been identified, namely the residues near the breaking loops in TM4 (Ala229, Trp232, Leu236) and TM10 (Met876, Leu879). As the breaking loops are considered to have an important function in the drug-translocation pathway by acting like flexible hinges that open or close a gate region, interaction with these residues could help to keep the gate closed and prevent the ligands from re-entering the intracellular space. In addition, the residues in the second third of TM1 (Leu65, Met68), TM11 (Gln946, Met949, Tyr950, Tyr953) and TM12 (Ala987), and the residues in the last third of TM5 (Tyr307), TM3 (Gln195), TM6 (Ser344) and TM7 (Phe728, Phe732) interact uniquely with the active compounds.

A significant difference in the number of interactions within the drug-binding site between active and non-active compounds was also observed (Table 1). The P-gp ligands formed a greater number of interactions within the binding pocket, along with at least one hydrogen bond contact, whereas the non-active compounds had fewer interactions and almost no presence of hydrogen bonds (see Supplementary Materials, Figure S3). The number of non-bonded interactions for the active compounds ranged from 13 to 27, and occurred mainly with aromatic and hydrophobic residues. Hydrogen bonding was also more frequent in the active compounds occurring mainly with tyrosine residues. Hydrophobic and hydrogen-bonding interactions are crucial in ligand–P-gp binding, as a large number of these interactions correlate with a high affinity for the protein [34,35]. 

As the smaller molecules in the group have a lower number of interactions with the receptor, the size of the ligand might be related to the number of interactions formed. However, the formation of hydrogen bonds seems to be independent of the molecule size, e.g., AMI and APD form only one hydrogen bond with P-gp, even though they have a large difference in their size and number of hydrogen bond donors (HBD) and hydrogen bond acceptors (HBA) (see Supplementary Materials, Table S2).

**Figure 5:**
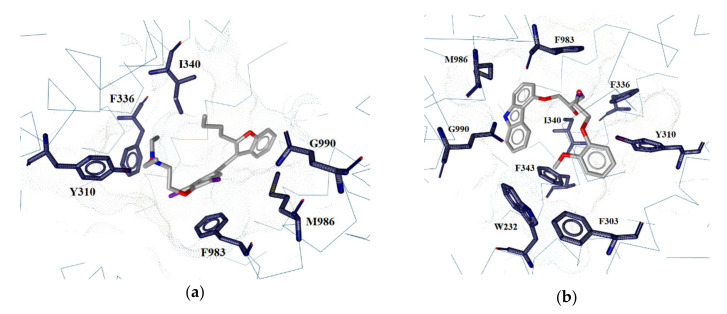

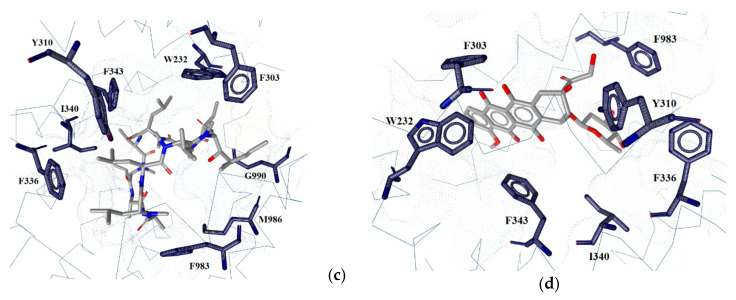
Simultaneous interactions detected in the active compounds: (**a**) amiodarone, (**b**) carvedilol, (**c**) cyclosporine A, (**d**) doxorubicin.

Simultaneous interactions with Tyr310, Phe336, Ile340, and Phe983 are present in all of the active compounds and none of the inactive ones, indicating that they may be crucial for the activity of the compounds. Furthermore, several other residues were identified that interact simultaneously with at least three active compounds, including Gln990 and Met986, interacting simultaneously in P-gp–AMI, P-gp–CAR and P-gp–CSA systems, and Phe343, Phe303 and Trp232, interacting simultaneously in P-gp–CAR, P-gp–CSA and P-gp–DOX systems (Figure 5). Some residues seem to be essential for ligand binding, and aromatic and/or hydrophobic contacts may be a key feature that determines the binding affinity of substrates and inhibitors within the binding pocket. 

Looking at the available experimental data on the residues involved in the binding of co-crystallized ligands, a high level of agreement is observed: 74,2% of the identified interacting residues correspond to residues experimentally found to be involved in substrate or inhibitor binding to P-gp [11,36,37], demonstrating the consistency between the MD simulation results and the available co-crystallized/cryoEM data.

**Figure 6:**
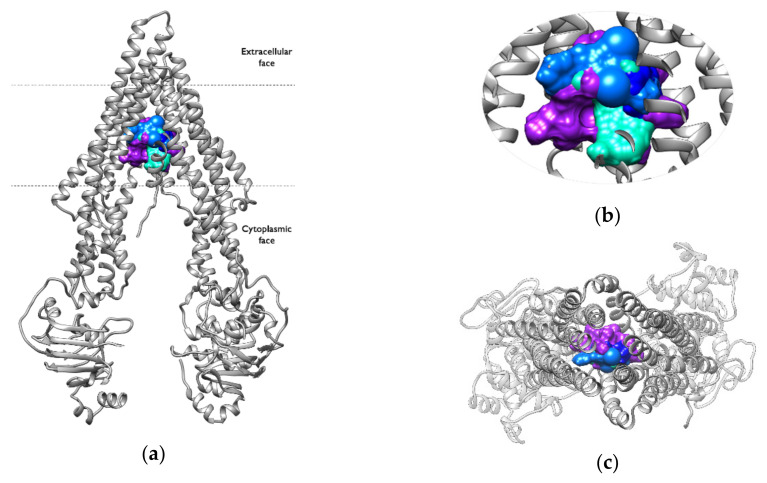
Interaction region of the active compounds within the binding pocket: (**a**) frontal view, (**b**) zoomed view, (**c**) view from the extracellular side of the protein looking into the inner chamber. The ligands are shown in surface representation; AMI is shown in lighter blue, CAR is shown in blue, CSA is shown in purple, and DOX is shown in turquoise.

**Figure 7:**
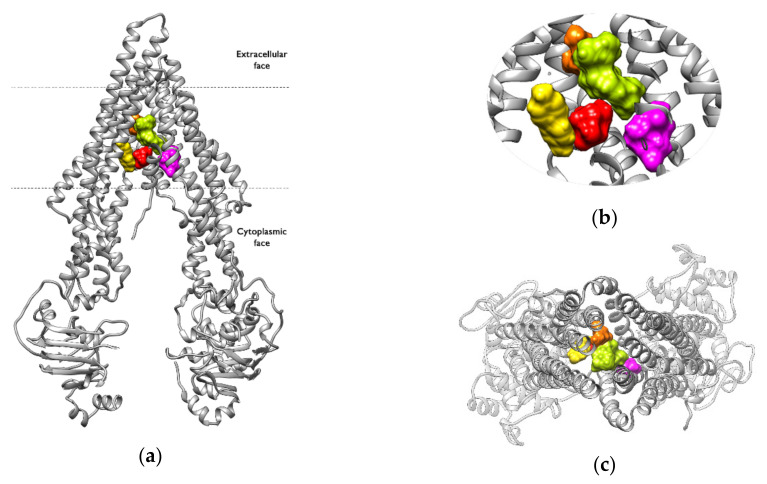
Interaction region of the non-active compounds within the binding pocket: (**a**) frontal view, (**b**) zoomed view, (**c**) view from the extracellular side of the protein looking into the inner chamber. The ligands are shown in the surface representation: APD is shown in red, BUS in is shown magenta, GEN is shown in light green, PQT is shown in yellow, and VPA is shown in orange.

Figure 6 and Figure 7 show the interaction regions within the binding pocket for each studied system. The centroids of each cluster, as obtained from the cluster analysis of the production trajectories, were used to determine the position of the compounds in the binding pocket. All of the active compounds share a common interaction region with a large overlap in the molecular surface (Figure 6). The interactions were established in a central region of the binding pocket in all of the clusters, indicating a stable binding during the simulation time. 

**Figure 8:**
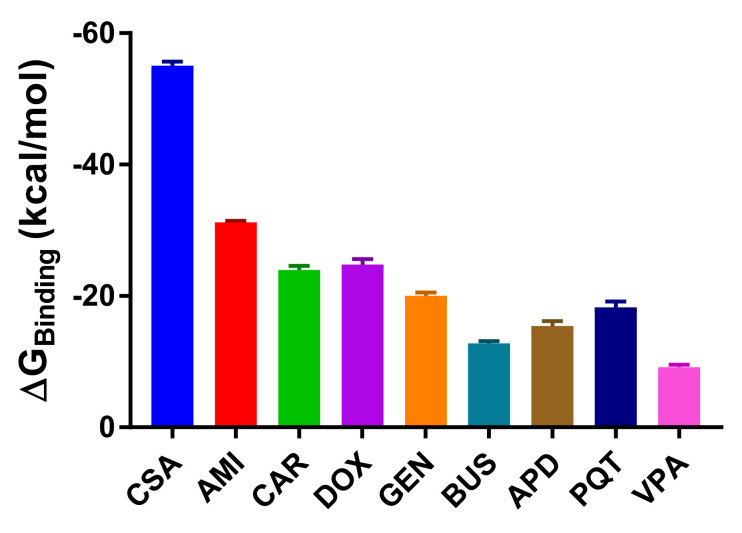
Bar graph of the estimated free energy of binding for the ligand–P-gp complexes studied using MM/PBSA calculations over 100 frames sampled from the entire 500ns trajectory (*n* = 100, mean ± SEM). Error bars: SEM.

In contrast, the non-active compounds were found to form interactions in different regions of the binding pocket (Figure 8), suggesting less stability in the binding mode, e.g., BUS, mostly interacting at a lower right site of the binding pocket, but in the second cluster the interactions occur at an upper left site (see Supplementary Materials, Figure S4). The greater conformational variability of the inactive compounds within the binding pocket might be related to physicochemical properties such as molecular weight or volume.

It should be noted that GEN is the inactive compound with the highest molecular weight in the group (Supplementary Materials, Table S2), and it has a similar number of contacts to some active compounds (Table 1). However, it does not interact with any of the amino acids linked exclusively to active compounds, nor does it show the simultaneous interactions observed with the active molecules. This compound is an example of how it is not enough to be “properly” oriented in the active site; specific interactions must also be present in order to be transported by P-gp.

In the study of P-gp ligand activity, the physicochemical properties of the compounds under investigation are another factor that should be considered (see Supplementary Materials, Table S2), as they play an important role in the determination of bioavailability under physiological conditions. Most of the non-active compounds in the group have negative logP values and are more hydrophilic, a physicochemical property that may prevent them from reaching the binding pocket. The high permeation rate of some compounds within the lipid bilayer is essential for the modulation of P-gp. This is due to a competitive mechanism for the binding site between inhibitors and substrates [38], but is not limited to modulation, as many of the substrates also have high logP values that promote permeation within the membrane.

### 2.3. Binding Free Energy Calculations

In the MM/PBSA calculations of the energies, the entropic term was neglected, as the current methods for entropy estimation are highly computationally intensive and inaccurate. For the purposes of this study, we were more interested in the relative free energies of binding, for comparison within the set of studied systems, rather than in the true free energy values. We should therefore bear in mind that the results presented here do not correspond to the real free energy values, as we have not estimated the entropy contribution to binding.

Across the simulations, all of the active compounds had lower—and consequently more favourable—estimated free energies of binding (Figure 8). CSA is the compound with the lowest estimated energy (−55.09 kcal/mol), and is thus the most stable in the group of studied systems. On the other hand, the small-molecule VPA has the highest and least favourable estimated free energy of binding (−9.15 kcal/mol), confirming the weak affinity of the compound and the low stability of the complex, which was also reflected in the low number of established contacts within the binding pocket (Table 1). The hypotheses that emerged from the analysis of the ligand–P-gp interactions in terms of the affinities of the compounds are further confirmed by the estimation of the binding free energies, and correlate with the available literature [23,39] and the experimental results of the transport assay in [19]. A full description of the energy components can be found in the Supplementary Materials, Table S3.

The dissociation constants (*K*_d_) were calculated based on the previously estimated free energy values, and were then compared with the available experimental data [40,41,42] (Supplementary Materials, Table S3). In both cases, we can conclude that CSA has the highest binding affinity, with *K*_d_ values of 0.2 μM (experimental) and 1.8 × 10^−34^ μM (calculated). The *K*_d_ values of the other active compounds also indicate that they bind more strongly to the target than the inactive ones. Although the calculated *K*_d_ values differ in magnitude from the experimental data (as the calculations are based on a very approximate method of the estimation of the binding free energy), they are still very useful for comparison and ranking purposes.

### 2.4. Structural Analysis

**Figure 9:**
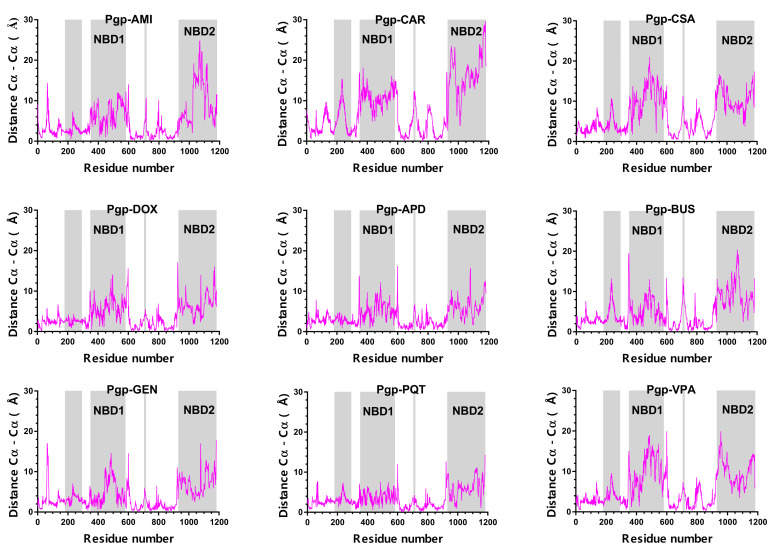
Cα distance between the centroids of the most-populated cluster in each system and the cryoEM structure of the *h*P-gp (PDB ID: 6QEX). The residue numbers do not correspond to the numbers in the PDB file of the cryo-EM structure, but are consecutive, as required by the simulation software.

The centroids of the most populated cluster in each system, obtained from the cluster analysis of the production trajectories, were used to analyse the significant changes in the protein structure. For this purpose, the Cα-distance between each centroid and a reference structure (cryoEM structure PDB ID: 6QEX) was measured. The largest deviations in the structure of all of the systems occured in the region of the NBDs, and to a lesser extent in the region of TM4 and TM5, and in the loop region between TM8 and TM9 (Figure 9). As the largest deviations occured in the NBDs, the distance between NBD1 and NBD2 was monitored, measured as the separation between the backbone nitrogen in the Lys residue of the Walker A motif in NBD1 and the Cα of the Ser residue in the signature motif of NBD2, conserved motifs which are important for ATP binding and hydrolysis [43]. Large relative motions were observed between the NBDs which were more marked in the active systems (see Supplementary Materials, Figure S5).

**Figure 10:**
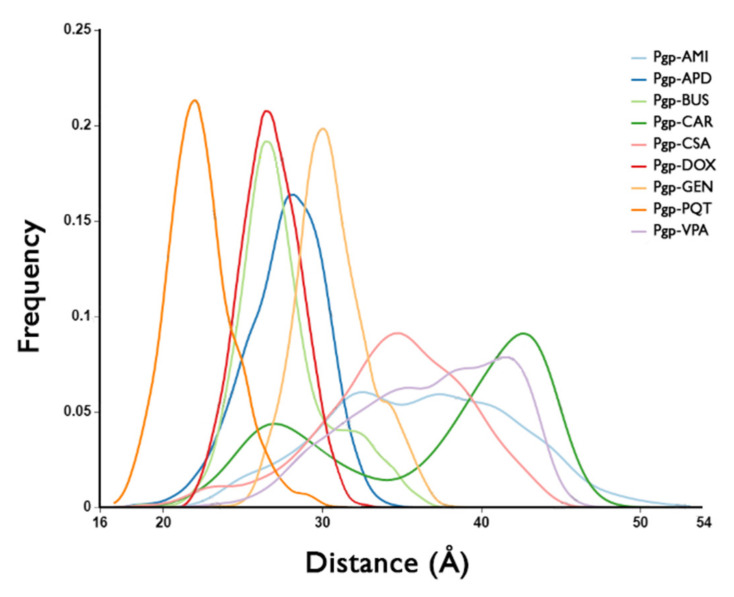
NBDs’ distance distribution curves over all of the trajectories for the studied systems.

The conformational differences between the active-bound and non-active-bound states can be better observed in the distance distribution curves of the NBDs (Figure 10). The broad distance distributions in the active-bound complexes indicate that these systems are highly dynamic despite the absence of nucleotides. In contrast, the non-active bound systems show narrower distance distribution curves, demonstrating more limited conformational flexibility. The protein is in a less-active state, and tends to stabilise in a single conformation. It is noteworthy that DOX, an active ligand, has a similar curve to BUS, an inactive compound. This similarity indicates that the NBDs in this system have not yet reached the same dynamics as the other active complexes in the studied group during the simulated time of 500 ns, probably due to a difference in the transport kinetics of DOX compared to the other active molecules, i.e., the mechanism undergone might be slower and might require longer simulation times to observe a broader distribution, as in the other active complexes.

**Figure 11:**
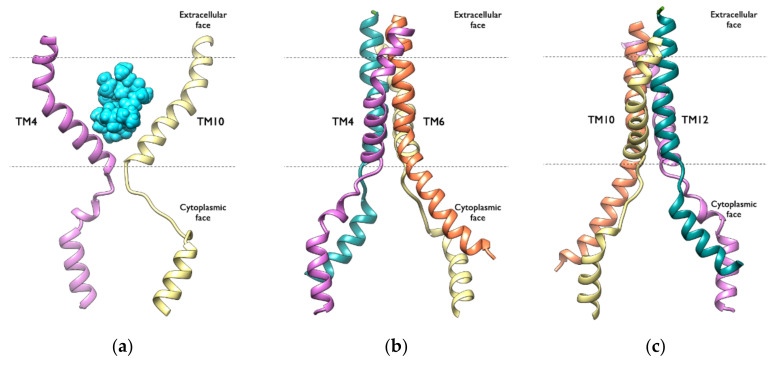
Ribbon representation of the TM segments which are important for the P-gp activity: (**a**) TM4 (pink) and TM10 (yellow) adopting a kinked conformation, with CSA located in the centre of the occluded cavity; (**b**) the TM4 (pink) and TM6 (orange) portal; (**c**) the TM10 (yellow) and TM12 (green) portal.

Previous studies have found that TM4 and TM10 shape a gate region which is open to the cytoplasm. Interestingly, in all of the simulated systems, the TM4 and TM10 segments adopt a kinked conformation that closes the cytoplasmic gate to the drug-binding site, forming an occluded cavity. During the simulation time, all of the studied compounds were found in the center of this cavity (Figure 11a). Moreover, the two portals formed by TM4/TM6 and TM10/TM12 (Figure 11b,c), which allow the access of hydrophobic molecules directly from the inner leaflet of the membrane, adopt the same conformation in all of the complexes studied: the two portals are open wide enough to accommodate hydrophobic molecules and allow P-gp to scan the inner leaflet to select and bind specific hydrophobic drugs prior to transport.

### 2.5. Concerted Motions in P-Glycoprotein

Principal Components Analysis (PCA) was used to determine the conformational changes and dominant modes of motion undergone by the P-gp during the simulation time. The covariance matrix of the 1,182 alpha carbons in the protein was calculated and diagonalized to obtain the eigenvectors and eigenvalues describing the motion of the system. The first two principal axes, PC1 and PC2, together explain between 44.49% and 71.55% of the total system motion (Table 2). The trajectories were then projected on PC1 (the PC that captures the most structural variance along the trajectory) and the PCs explaining at least 85% of the total flexibility of the complexes.

**Figure 12:**
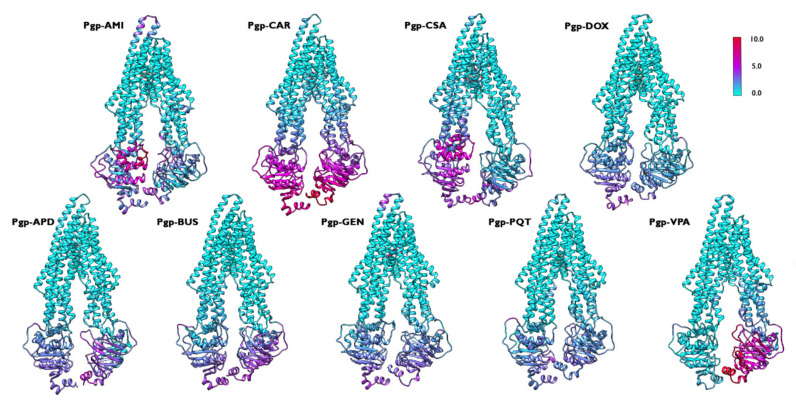
Cα-RMSF coloured representation for the simulated P-gp–ligand systems along the first principal component (PC1) calculated from the 500 ns production run. The flexibility scale goes from cyan (lower values) to red (higher values).

Along the first principal component (Figure 12), most of the conformational changes of the active-bound complexes are associated with NBD1, except for P-gp–CAR, where both NBDs carry most of the flexibility. On the other hand, the conformational changes of the non-active bound systems are less wide, and are more evenly distributed between both NBDs. Only the NBD2 carries most of the flexibility in the P-gp-VPA complex. This behaviour—which was already noticed during the analysis of the backbone RMSF, and was also observed here in the analysis along the PC1—could be attributed to the additional property of VPA to affect the expression and function of P-gp, as explained in Section 2.1.

Regarding the atomic fluctuations along the PCs, which explain at least 85% of the conformational changes, the flexibility is concentrated in both NBDs for all of the systems, which is similar to the RMSF results discussed in Section 2.1 (see Supplementary Materials, Figure S6).

The analysis of the single atomic fluctuation values along the first principal component showed that the residues of the ABC motif in NBD1 have higher fluctuations in each active-bound system than in any of the other systems, with flexibility increases of 6.08% to 13.42% compared to the non-active complexes. This variation is not visible when analysing the PCs that explain at least 85% of the motion of the complexes. The fact that the residues of the ABC motif, which are involved in ATP binding, are more flexible in the active-bound systems, and that this flexibility is observed only in the ABC motif of one NBD, suggests some asymmetry in the motion pattern of the NBDs when the protein is bound to an active compound.

The structural variance of the transporter in the presence of an active compound along PC1 is dominated by the movement of the NBDs approaching each other with a large amplitude of motion, specifically for the complexes formed with AMI, CAR and CSA. Although the type of motion in each system is different (Supplementary Materials, Figure S7), the ABC motif in NBD1 and the Walker A motif in NBD2 tend to approach as the NBDs move. These two conserved motifs form the binding site for the nucleotide required to initiate the conformational changes which are characteristic of the transport cycle. In addition, the motion of the NBDs is coupled with the rotation of TM4, TM6, TM10, and TM12, which slightly open the binding pocket when the NBDs come together.

The structural variance of the non-active-bound complexes was also determined by the movement of the NBDs, but was characterised by a pendular motion perpendicular to the bilayer plane and narrower amplitudes (Supplementary Materials, Figure S8) with no tendency to approach to each other, indicating a less flexible and active system in which the conformational changes associated with the transport cycle do not seem to be initiated.

Interestingly, the direction and amplitude of the NBDs’ movement in the P-gp-DOX system (Supplementary Materials, Figure S9) are more similar to the pattern followed by the non-active compounds, suggesting a different transport mechanism for this ligand. The transport process may be slower, and therefore longer simulation times would be required to observe the same concerted movements as in the other active systems.

### 2.6. Binding Pocket

It is important to evaluate the ways in which conformational changes in the TM helices might affect the volume of the internal cavity. To this end, the volume of the binding pocket for each system was calculated during the MD simulations using POVME 3.0.

**Figure 13:**
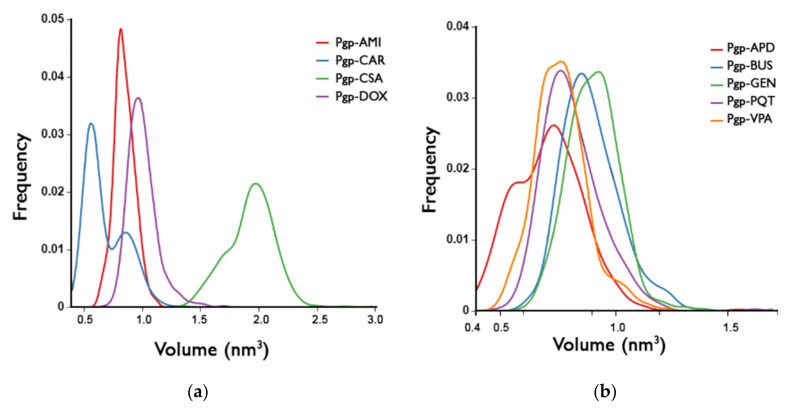
Distributions of the internal cavity volumes for (**a**) active-bound systems and (**b**) non-active-bound systems.

In all of the systems studied, the binding pockets exhibit a broad distribution of volumes along the simulation, with no significant differences or patterns in the volume distribution between active- and non-active-bound systems (Figure 13). The largest volumes observed correspond to CSA, which is the largest molecule in the group, demonstrating the ability of the binding pocket to adapt to the size of the ligand. However, the values of the volumes for the non-active systems are in the same range as those of the active ones, even though these compounds are the smallest in the group, and do not induce conformational changes that reduce the volume of the binding pocket. This lack of induced-fit capability—probably due to the weak interactions with the receptor—could be related to the inability of the protein to transport these compounds.

A cluster analysis of the binding pocket volumes was performed on the combined trajectories of the active-bound and non-active-bound systems, separately. Eight clusters were generated from the active-bound systems, and twelve clusters were obtained from the non-active-bound systems. Each analysed frame was assigned to a single cluster representing frequently visited pocket shapes. For the active systems, each cluster consisted of members of a single active-bound system, indicating that the binding pocket shapes of these complexes differ from each other. In contrast, for the non-active-bound systems, each resulting cluster was formed by members of multiple systems; therefore, the pocket shapes are very similar between the complexes. In the Supplementary Materials, in Figures S10 and S11, can be observed the average pocket shape of the active and non-active systems, as well as the areas where each cluster opens or closes more than the average shape.

### 2.7. Exposure of Surfaces to the Solvent

**Figure 14:**
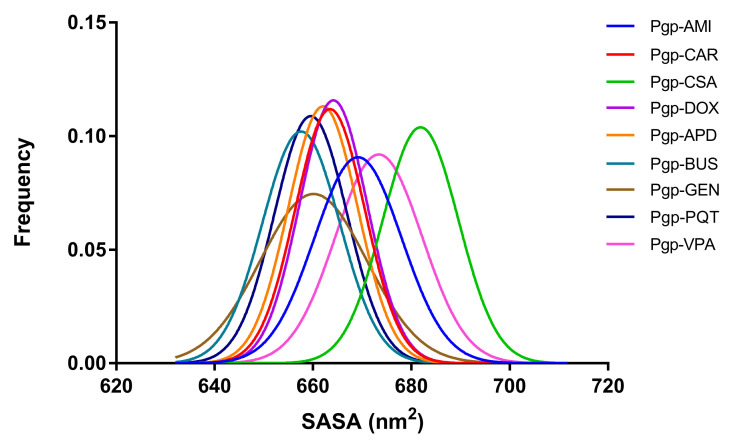
Distributions of the total solvent-accessible surface area (SASA) for the studied systems.

The changes in the solvent accessibility of the protein can be determined by calculating the solvent-accessible surface area (SASA). The total SASAs of the different P-gp systems was determined using the CPPTRAJ module [44] in AmberTools [45], and are shown in Figure 14, where smaller peak values are observed for the non-active-bound systems. Smaller SASA values indicate that the hydrophobic core is more protected from the external environment; it could shrink, resulting in more-compact and less-flexible systems. This reduced flexibility may have a significant impact on the structural conformation of the protein, and may consequently affect its functional behaviour.

The greater flexibility of NBD2 in the VPA system, as noted in the RSMF and principal component analyses, was also confirmed by the larger SASA values observed during the simulation time. As mentioned earlier, VPA is an inactive compound that is not transported by and does not inhibit P-gp, but which plays a different role with the protein, namely as a trigger for its expression and function, with this being a possible reason why the behaviour of this system in certain analyses is not consistent with that of the other complexes in the study.

**Figure 15:**
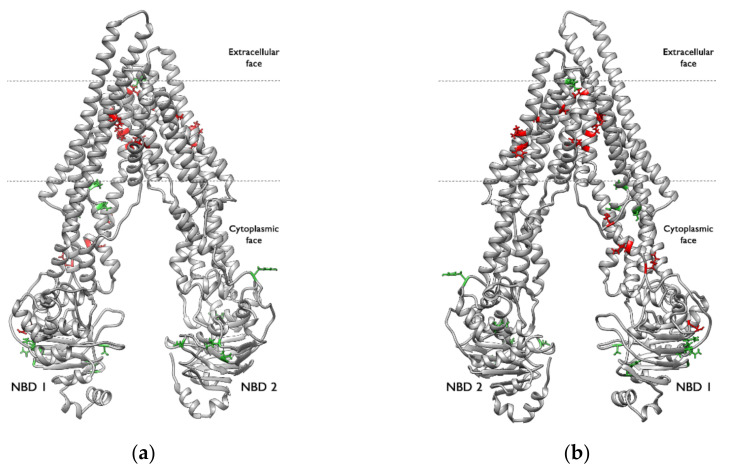
P-Glycoprotein residues with significant variations in the solvent-accessible surface area (SASA): (**a**) front view and (**b**) back view. The residues coloured red have a smaller SASA in the systems formed by P-gp and an active compound. The residues coloured green have a larger SASA in the systems formed by P-gp and an active compound.

The SASA values for each residue were calculated using the Python library MDtraj [46]. Some residues located in the TM region have smaller average SASA values in each active-bound system than those in any of the other systems (Figure 15, Supplementary Materials, Table S4). In particular, residues M69, I340, F343, Y953, and M986, which are involved in non-bonded contacts, show the largest difference (see Supplementary Materials, Table S4). These residues are more buried and less flexible in the active-bound systems, probably due to the strong hydrophobic interactions that stabilize the ligands. On the other hand, there are some residues in the NBDs that have larger average SASA values in each active-bound system than in any of the other systems (Figure 15, Supplementary Materials, Table S5). These residues are more exposed to the solvent, confirming the higher flexibility of these domains when there is an active ligand in the binding pocket. They are less buried and better able to explore the conformational space, which may lead to the motion patterns analysed in Section 2.5.

## 3. Materials and Methods

### 3.1. Preparation of the Initial Structures

The initial structure of P-gp used for the simulations corresponds to the cryoEM structure of the *h*P-gp (PDB ID: 6QEX) [11]. The starting configuration for the MD simulations of the ligand–P-gp complexes was obtained from previous docking calculations performed using the CDOCKER algorithm [47] within the Dock Ligands protocol in Discovery Studio 4.1 [48], as reported in [19]. The starting complexes were parametrized using the CHARMM36 force field through the CHARMM-GUI web-based graphical user interface [49,50].

### 3.2. Systems Construction

After the parametrization of the ligand–P-gp complexes, the systems were partially solvated using the TIP3P explicit water model with the software DOWSER [51] and SOLVATE [52]. The water molecules located in the hydrophobic region of the protein were then removed. The partially solvated systems were embedded into a 1-palmitoyl-2-oleoyl-phosphatidylcholine (POPC) membrane patch constructed using the membrane plugin from VMD [53] and parametrized using the CHARMM27 force field. The system was placed in the center of the POPC lipid bilayer, with its long axis perpendicular to the lipid surface. Finally, the entire system was solvated using VMD’s Solvate plugin in order to generate the water simulation box. Potassium (K^+^) and chloride (Cl^−^) ions were then added at a concentration of 0.2 M to neutralize the system using VMD’s Autoionize plugin.

### 3.3. Molecular Dynamics Simulations

As the MD simulations were performed using Amber 2018 software [45], the tool CHAMBER [54] was employed to convert the CHARMM files into the AMBER format, and to enable the use of the CHARMM force field within the AMBER’s MD engines.

#### 3.3.1. Simulation Parameters

Periodic boundary conditions (PBC) were applied in all of the simulations. The bonds involving hydrogen atoms were constrained using the SHAKE algorithm. The long-range electrostatic interactions were estimated using the Particle-Mesh Ewald (PME) method, and the non-bonded cutoff radius for the van de Waals and electrostatic interactions was set to 10 Å. The temperature of the system was equilibrated at 303 K with a collision frequency of 1.0 ps^−1^ using the Langevin thermostat. For the NPT runs, the pressure was maintained at 1 bar using the Berendsen barostat. The system pressure was semi-isotropically coupled, i.e., the pressure was balanced by separately coupling the lateral (x and y) and normal (z) box directions. The CPPTRAJ [44] module of AmberTools [45], VMD [53], UCSF Chimera [55], MDtraj [46], and POVME 3.0 [56] were used for the trajectory analyses.

#### 3.3.2. Energy Minimization

An energy minimization run of 6000 cycles was performed in three successive steps of 2000 cycles each using the steepest descent method: first, restrains were applied to the whole system with a force constant of 100 kcal/mol Å^2^, with only the water molecules moving freely, followed by a second cycle with restrains applied only on the ligand and protein. In the third step, the entire system was minimized without the application of any restrains.

#### 3.3.3. Heating

After the minimization steps, the system was gradually heated to 303 K in the NVT ensemble for 1 ns, applying a restraining force of 100 kcal/mol Å^2^ to the protein, ligand, and POPC lipid bilayer. The heating was performed in two steps: in the first 500 ps, the system was heated from 0 to 150 K, and in the last 500 ps it was heated from 150 K to 303 K.

#### 3.3.4. Equilibration

The equilibration phase was performed in the NPT ensemble with a restraining force of 100 kcal/mol for a total of 30 ns, divided into five steps: first 20 ns with restrains on the protein, ligand and POPC lipid bilayer, then 2.5 ns with restrains on the protein and ligand. Finally, three consecutive 2.5 ns NPT runs were performed in order to progressively remove the restrains on the protein: first with restrains on the protein except Cβ, then with restrains on the proteins except Cα and Cβ, followed by a final 2.5 ns equilibration run without any restrains. This equilibrated system was the starting point for the 500 ns completely unrestrained NPT production run.

#### 3.3.5. Production

The production phase was performed in the NTP ensemble at a temperature of 303 K and a pressure of 1 bar for 500 ns. The time step was set to 2 fs, and the trajectories were saved every 90,000 steps (180 ps).

### 3.4. Trajectory Analysis

In order to analyse the average deviations in the atomic positions and the stability of the MD trajectories, the atomic root-mean-square deviations (RMSD) of each system were calculated using the CPPTRAJ module [44] of the AmberTools package [45]. For the RMSD calculation, each frame of the trajectory was aligned along the protein backbone relative to the initial structure.

#### 3.4.1. Binding-Free Energy Calculations

The free energies of binding of the ligand–P-gp complexes studied were estimated using the Molecular Mechanics/Poisson-Boltzmann Surface Area (MM/PBSA) method [57], based on 100 frames extracted from each trajectory and selected by clustering analysis. Only the heavy atoms of the ligands were considered for the clustering. The representative MD snapshots were selected as the centroids of 100 clusters resulting from the grouping of all of the ligand conformations obtained from the production runs. The k-means algorithm, as implemented in CPPTRAJ, was employed for the cluster analyses.

The MM/PBSA calculations were performed using the Python script MMPBSA.py [58] implemented in Amber 2018, and the parameters used were set as follows: the lipid membrane was treated implicitly; therefore, the POPC lipid bilayer, water molecules, and ions were removed from the trajectories. The solute dielectric constant was set to 4, and the implicit solvent dielectric constant was set to 80. A heterogeneous membrane dielectric constant varying from 1 in its centre to 80 in the edge of the membrane (memopt=3) was used. The ionic strength was set to 0.15 M.

The binding free energies are calculated by subtracting the free energies of the unbound receptor and the ligand from the free energy of the bound complex, as shown in Equation (1).

(1) $$\Delta G_{binding}=\Delta G_{Complex}-\left(\Delta G_{Protein}+\Delta G_{Ligand}\right)$$

The free energy change associated with each term for the protein, ligand and complex is estimated according to Equation (2):

(2) $$\Delta G=E_{Gas}+\Delta G_{Solvation}-TS_{Solute}$$

where the $E_{Gas}$ term includes the sum of the bonded and non-bonded interaction energies (*V^vdw^* and *V^ele^*). The gas-phase energies are often the molecular mechanical (MM) energies from the force field, while the solvation-free energies ($\Delta G_{Solvation}$), which include *G^polar^* and *G^non-polar^* contributions, are calculated using an implicit continuum solvent model, representing the change in free energy due to the conversion of a solute in a vacuum to its solvated state. In MM/PBSA, *G^polar^* is obtained by solving the Poisson–Boltzmann equation, while the *G^non-polar^* term is often approximated by a term of the solvent-accessible surface area (SASA). *T* and *S* represent the temperature of the system and the entropy of the solute in vacuum. The *TS* terms, which enter Equation (1) via the individual ΔG terms, account for the change in conformational entropy of the ligand–protein complex upon ligand binding, and are estimated using known approximations, e.g., the Normal Mode Analysis of the protein–ligand coordinates resulting from the simulation of the complex. Often, the entropic term is neglected because there is more interest in the relative free energies of binding for comparison with similar systems than in the true free-energy values. In addition, the current methods for entropy estimation are highly computationally intensive and inaccurate.

The energies described in the equations above are the single-point energies of the system. However, in practice, these energies are calculated according to averages from an ensemble of representative structures. The expression of Equation (2) in terms of averages yields Equation (3):

(3) $$\Delta G\;≅\langle E_{Gas}\rangle +\langle \Delta G_{Solvation}\rangle -\langle TS_{Solute}\rangle \;=\frac{1}{N}\overset{N}{\underset{i=1}{\sum }}E_{i,Gas}+\frac{1}{N}\overset{N}{\underset{i=1}{\sum }}G_{i,Solvation}-\frac{T}{N}\overset{N}{\underset{i=1}{\sum }}S_{i,Solute}$$

where *i* is the index of the frame and *N* is the total number of frames analysed.

The dissociation constant (*K_d_*) of each system was also calculated based on the estimated free energy values according to the following equation:

(4) $$\Delta G=-\mathrm{RTln}\left(K_{d}\right.)$$

#### 3.4.2. Ligand–Protein Interactions

The frequencies of the ligand–protein interactions were explored on the 500 ns production trajectories of each system using the structureViz2 Cytoscape plugin [59] and UCSF Chimera software [55]. The analysis of the relevant interactions in each system was performed based on the interactions observed in at least 50% of the snapshots.

#### 3.4.3. Clustering Analysis

Clustering analysis was performed on the 500 ns production trajectories of each system using the *Cluster* analysis command of the CPPTRAJ module [44] in the AmberTools package [45], with the goal of determining the structural populations from the simulated trajectories. Clustering is a way of partitioning data such that the data points within a cluster are more similar to each other than to points outside a cluster, i.e., similar conformations are grouped together. The similarity between the members of a cluster is determined by a distance metric, usually the coordinates RMSD.

A number of clusters—ranging from two to twenty—was analysed for each system studied, and the behaviour of the metrics DBI, pSF, and SSR/SST [60] was examined to determine the optimal number of clusters for each system. The DBI and pSF values are metrics of clustering quality; low DBI and high pSF values indicate better results. On the other hand, the R-squared value (SSR/SST) represents the percentage of variance explained by the data. Theoretically, the optimal number of clusters is reached when the DBI has a minimum, the pSF shows a maximum, and the SSR/SST plot reaches a plateau. These conditions are difficult to satisfy simultaneously in a real clustering problem; thus, a balance between them was made to select the optimal number of clusters. The corresponding plots and the number of clusters selected can be found in the Supplementary Materials, Figure S12.

#### 3.4.4. Principal Component Analysis

A principal component analysis (PCA) was performed to analyse the conformational changes and dominant modes of motion of P-gp during the 500 ns production run. PCA is a method used to transform a set of potentially coordinated observations into a set of orthogonal vectors, i.e., the principal components (PCs). The PCs explain the variance in the data, with the first PC carrying the largest variance, the second PC the second largest, and so on.

The input to the PCA was the covariance matrix calculated from the time series of the position coordinates, such that the PCs represent specific modes of motion of the system, with the first PC representing the dominant motion. The entries of this matrix are the covariance values between the X, Y and Z components of each atom, such that the final matrix has a size of 3N × 3N, where N is the number of atoms. As we are only interested in the internal dynamics of the system, the rotational and translational movements were removed by performing a coordinate RMS fit to a reference structure, i.e., the *h*P-gp cryoEM structure. After the removal of all of the translations and rotations, the covariance matrix was constructed based on the Cα coordinates.

The covariance matrix was then diagonalized to obtain the eigenvectors (PCs) and the eigenvalues (the weight of each PC) describing the principal modes of structural variation. The diagonalization process results in an orthogonal set of unitary vectors describing the directions of maximum variation in the observed conformational distribution. Each eigenvector is associated with an eigenvalue that determines the amplitude of the movements along each principal axis. The sum of all of the eigenvalues is considered to be the total conformational variance of the system, and then the relative contribution of each eigenvector to the total system flexibility is calculated as the ratio between its associated eigenvalue and the total system flexibility. PCA is useful for gaining insight into the dynamics of a system, but it should be kept in mind that the actual motion of the system over the course of a simulation is almost always a combination of the individual PCs.

PCA was performed using the CPPTRAJ module of the AmberTools package, and the visualization of principal component data was carried out using the Normal Mode Wizard (NMwiz) plugin [61] for VMD.

#### 3.4.5. Binding Pocket Volume

The binding pocket volume calculations were performed using the tool Pocket Volume Measurer POVME 3.0 [56]. The POVME algorithm calculates the pocket volume by subtracting the volume occupied by the protein atoms in each frame from the defined inclusion region, with this region defining the boundaries of the pocket. The analysis of the binding pocket volume for each system was performed on the input PDB trajectories containing 462 frames extracted from each individual production trajectory. The pocket inclusion region was ligand-defined, i.e., using the ligand residue name, the pocket was defined in all of the grid points within 3 Å of the ligand atoms in the loaded PDB trajectory.

In order to find representative binding pocket conformations, and to determine the average pocket shape in each group of systems, a pocket shape clustering procedure was also performed. For this purpose, the ligand was removed from the trajectories, and they were combined to obtain two new sets of trajectories: one with the combined trajectories of the active-bound systems, and one with the combined trajectories of the non-active-bound systems. The trajectories were aligned, and the inclusion region was geometrically defined using x, y, z coordinates and a radius value to define the location of the binding pocket.

The clustering of the pocket shape is completed in two steps: first, the similarity matrix of the binding pockets of all of the analysed frames is calculated, followed by the clustering of this similarity matrix. The similarity matrix was calculated using the Tanimoto overlap score of each pocket pair. The Tanimoto score of a pair of frames ranges from 0 (the two pockets have no volume in common) to 1 (the two pocket shapes are identical). The similarity matrix was clustered using the hierarchical clustering method, and the resulting average pocket shape of each group was visualized in VMD.

#### 3.4.6. Solvent-Accessible Surface Area (SASA)

The total SASA was calculated from the 500 ns production trajectories of each system using the Surf action command of the CPPTRAJ module [44] in the AmberTools package [45], which calculates the surface area in Å^2^ using the Linear Combinations of Pairwise Overlaps (LCPO) algorithm [62].

The per-residue SASA, on the other hand, was calculated using the Python library MDtraj [46].

## 4. Conclusions

This study provides evidence that the conformational distribution and dynamics of the NBDs differ when active or inactive compounds are bound to P-gp. The different behaviour is reflected in the motion patterns and structural variations undergone by the protein during the simulation time. In general, the complexes formed by P-gp and an active compound show the higher flexibility of the NBDs, with most of the conformational changes being associated with NBD1. Interestingly, even in the absence of the ATP molecule, the nucleotide binding site showed remarkable activity only in the active-bound complexes. This result may be of great use in the analysis of compounds with unknown activity for P-gp.

The binding cavity was able to adapt to the size of the molecule, and hence to accommodate ligands of different sizes. However, the size of the binding pocket remained larger than expected for the molecules that were not transported, leading to the formulation of the hypothesis that an important requirement for the transport of molecules by P-gp is their ability to induce changes in the binding pocket (the induced-fit model).

In some of the analyses performed, slight differences in the behaviour of the P-gp-VPA and P-gp-DOX complexes were observed. These variations could be attributed to different interaction mechanisms specific to these compounds, as the estimated free energies of binding confirmed their respective experimental P-gp affinities. Moreover, VPA is an inactive compound (it is not transported by and does not inhibit P-gp) that also plays another role with the protein, namely as an inducer of its expression and function. The inductive property of VPA could be a possible reason for the behaviour of this system in some of the analyses performed.

Molecular dynamics proved to be a sufficiently accurate method if we are to consider the flexibility of the target protein, as a high degree of agreement was found between the predicted and experimental data in terms of the interacting residues. However, considering that protein conformational changes can occur at least on the time scale of microseconds [63], it should be clear that our results are limited to the simulated time and conditions, e.g., the absence of the ATP molecule. Therefore, further studies involving the nucleotide molecule with longer simulation times are suggested in order to validate the results of this work.

## Figures and Tables

**Table 1:** Number and type of P-gp residues involved in non-bonded and hydrogen-bond contacts.

System	Simulation Contacts		Type of Residue		
	Non-Bonded	Hydrogen Bond	Aromatic	Aliphatic	Polar
AMI ^1^	16	1	8	8	5
CAR ^2^	11	2	6	5	2
CSA ^3^	25	2	8	17	9
DOX ^4^	12	1	6	6	2
APD ^5^	5	1	1	4	3
BUS ^6^	7	0	4	2	1
GEN ^7^	11	1	5	6	2
PQT ^8^	3	0	0	3	2
VPA ^9^	3	0	1	1	0

^1^ Amiodarone; ^2^ carvedilol; ^3^ cyclosporine A; ^4^ doxorubicin; ^5^ pamidronate; ^6^ busulfan; ^7^ gentamicin; ^8^ paraquat; ^9^ valproic acid.

**Table 2:** PCA analysis for the 500 ns simulation run of P-gp ligand-bound systems.

Eigenvector	Cumulated Eigenvalues Expressed in Percent (%) ^1^								
	AMI ^2^	CAR ^3^	CSA ^4^	DOX ^5^	APD ^6^	BUS ^7^	GEN ^8^	PQT ^9^	VPA ^10^
1	34.02	58.04	33.35	28.39	36.07	37.58	32.61	30.24	40.62
2	54.10	71.55	54.74	44.49	50.55	55.95	50.48	50.83	60.08
3	72.36	84.42	73.97	65.63	68.96	73.99	66.34	69.08	79.22
4	77.40	87.92	79.90	71.06	73.86	77.89	71.59	73.77	84.27
5	81.31	90.28	83.37	74.68	77.73	80.48	75.29	76.59	87.40
6	84.36	91.67	85.85	78.10	80.73	82.94	77.94	78.86	88.90
7	86.74	92.75	87.37	80.78	82.66	85.18	80.06	80.59	90.12
8	88.31	93.48	88.43	82.37	84.11	86.38	81.76	82.18	91.14
9	89.34	94.09	89.39	83.78	85.41	87.52	83.39	83.35	92.01
10	90.14	94.66	90.14	85.05	86.46	88.43	84.60	84.44	92.69
11	90.70	95.13	90.86	86.00	87.32	89.11	85.57	85.42	93.25
12	91.20	95.43	91.49	86.81	88.06	89.76	86.40	86.29	93.73

^1^ flexibility explained by eigenvectors from 1 to n; ^2^ P-gp–AMI; ^3^ P-gp–CAR; ^4^ P-gp–CSA; ^5^ P-gp–DOX; ^6^ P-gp–APD; ^7^ P-gp–BUS; ^8^ P-gp–GEN; ^9^ P-gp–PQT; ^10^ P-gp–VPA.

## Data Availability

Not applicable.

## Supplementary Materials

The following are available online at https://www.mdpi.com/article/10.3390/ijms23010362/s1.
