# Supplementary material for: Structure–Function Relationships in the Human P-Glycoprotein (ABCB1): Insights from Molecular Dynamics Simulations
Source: Int J Mol Sci. 2021 Dec 29;23(1):362. doi: 10.3390/ijms23010362 (PMC8745603; doi:10.3390/ijms23010362)
Supplement: Supplementary file 1 [file ijms-23-00362-s001.zip › ijms-1508388-supplementary.pdf]

Supporting Materials

Energy of the system

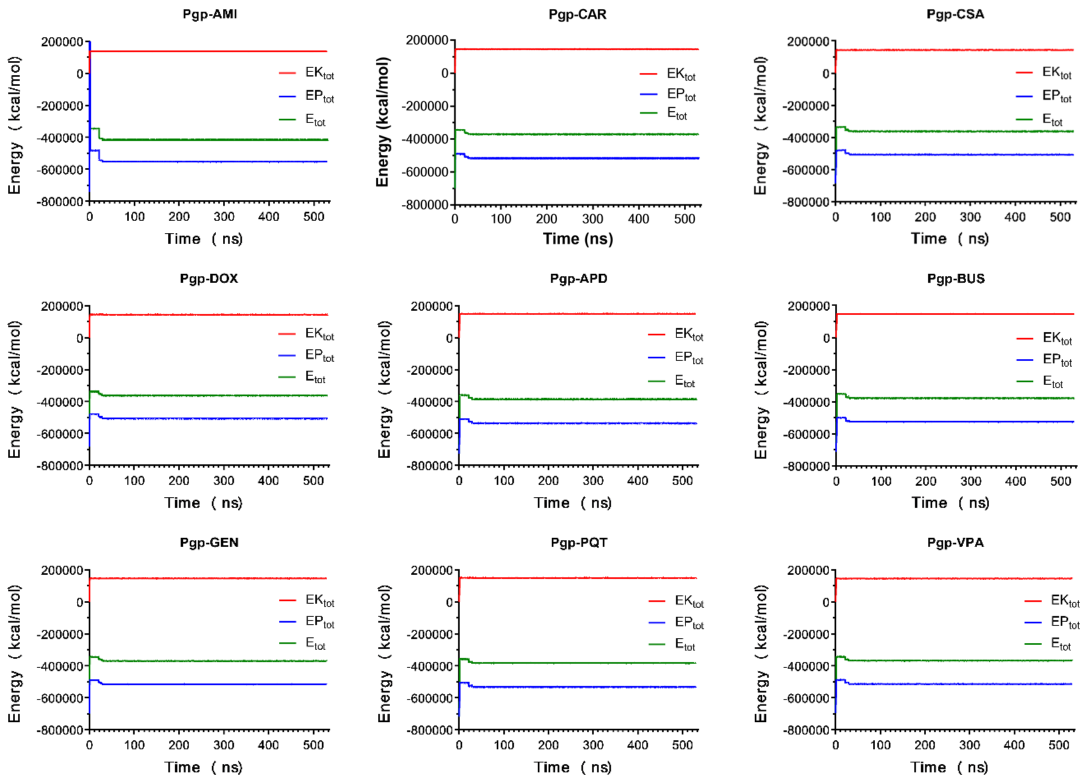

Figure S1: Energy of the simulated systems during the 500 ns production run. The red line shows the kinetic energy, and the blue line shows the potential energy. The green line shows the total energy.

Ligand–P-gp interactions

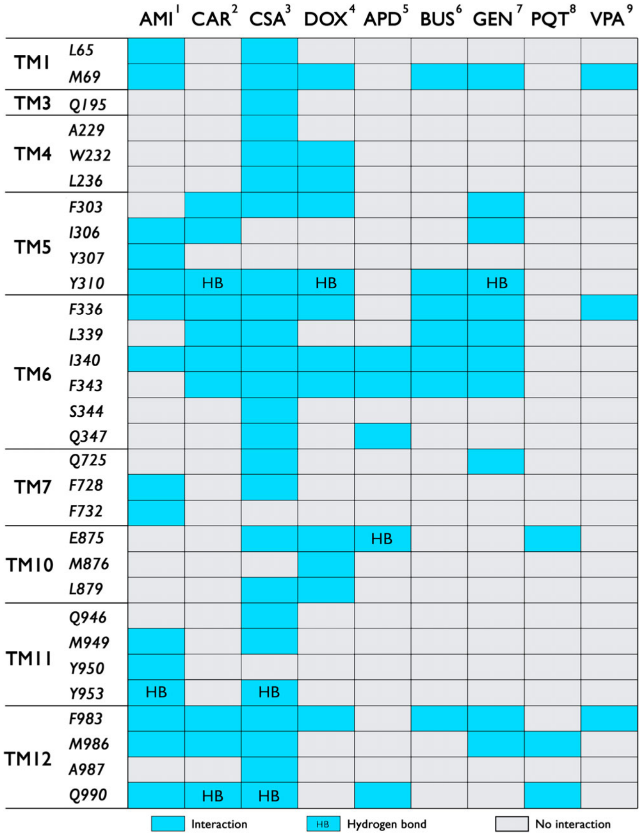

Figure S1: Ligand–P-gp interactions. Residues involved in non-bonded and hydrogen bond contacts. [1] Amiodarone; [2] carvedilol; [3] cyclosporine A; [4] doxorubicin; [5] pamidronate; [6] busulfan; [7] gentamicin; [8] paraquat; [9] valproic acid.

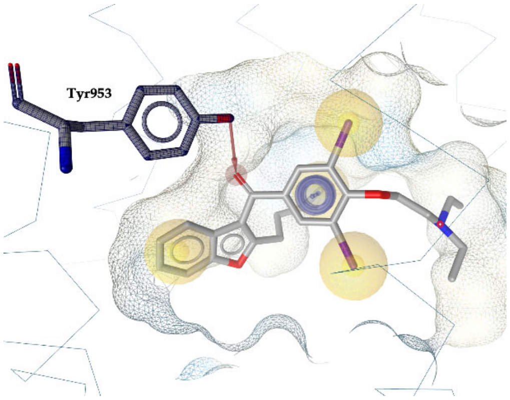

(a)

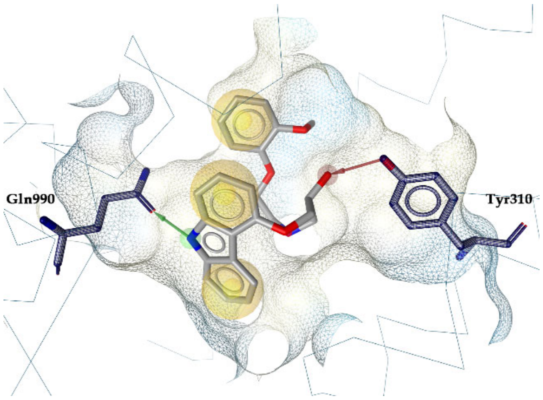

(b)

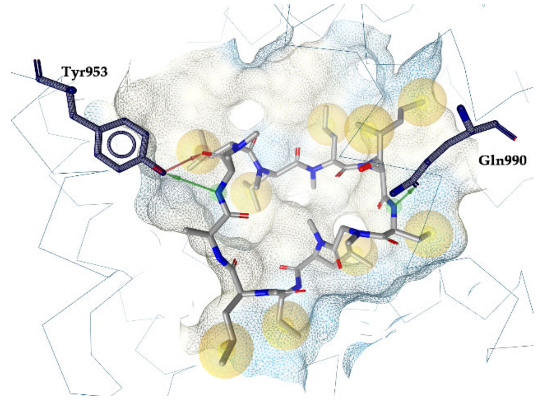

(c)

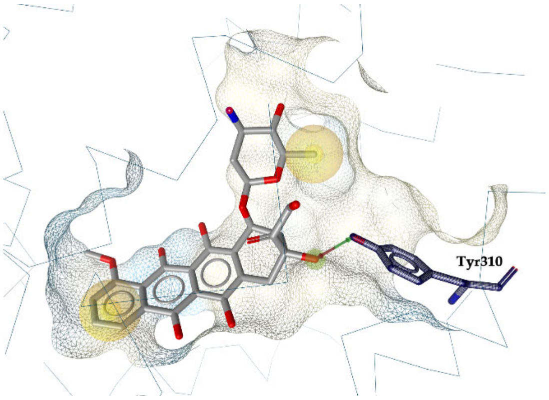

(d)

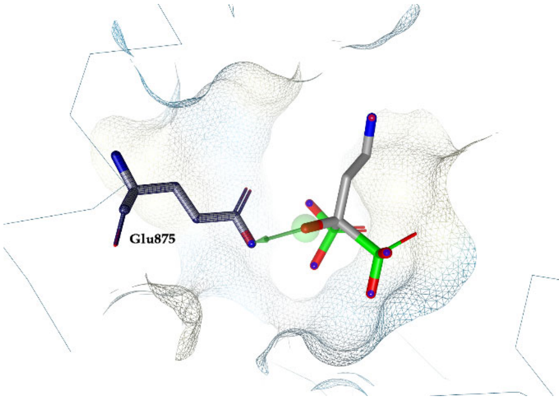

(e)

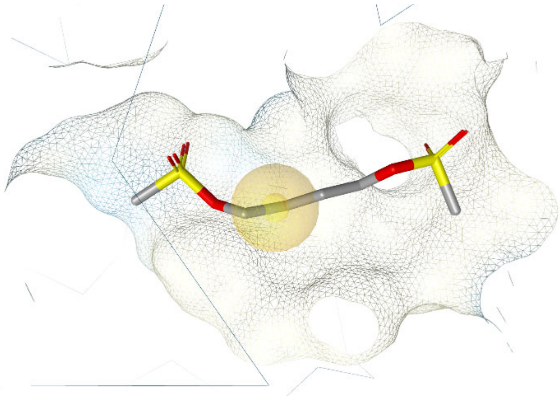

(f)

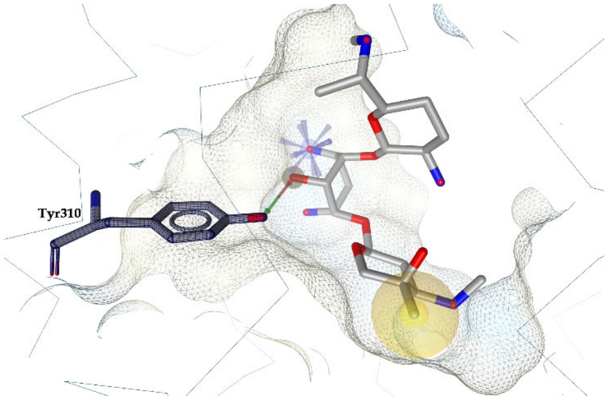

(g)

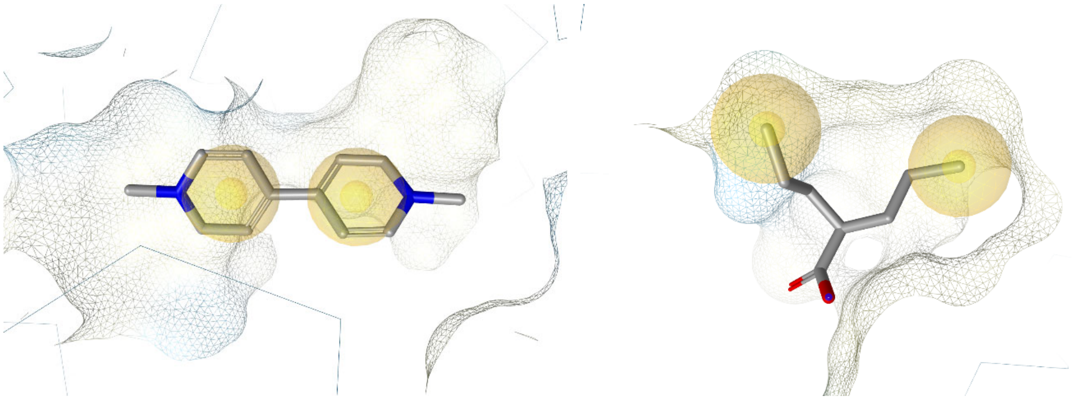

(h) (i)

Figure S3: 3D representation of the most relevant ligand–P-gp interactions within the binding pocket. (a) P-gp–AMI; (b) P-gp–CAR; (c) P-gp–CSA; (d) P-gp–DOX; (e) P-gp–APD; (f) P-gp–BUS; (g) P-gp–GEN; (h) P-gp–PQT; (i) P-gp–VPA. The binding pocket is shown in surface representation with a colour scheme corresponding to the hydrophobicity; non-polar regions are coloured in yellow. Residues involved in hydrogen bonding are exposed and highlighted with a dark blue mesh. Red arrows indicate hydrogen bond acceptor relationships, green arrows indicate hydrogen bond donor relationships, yellow spheres indicate hydrophobic interactions, and the blue ring indicates aromatic interactions.

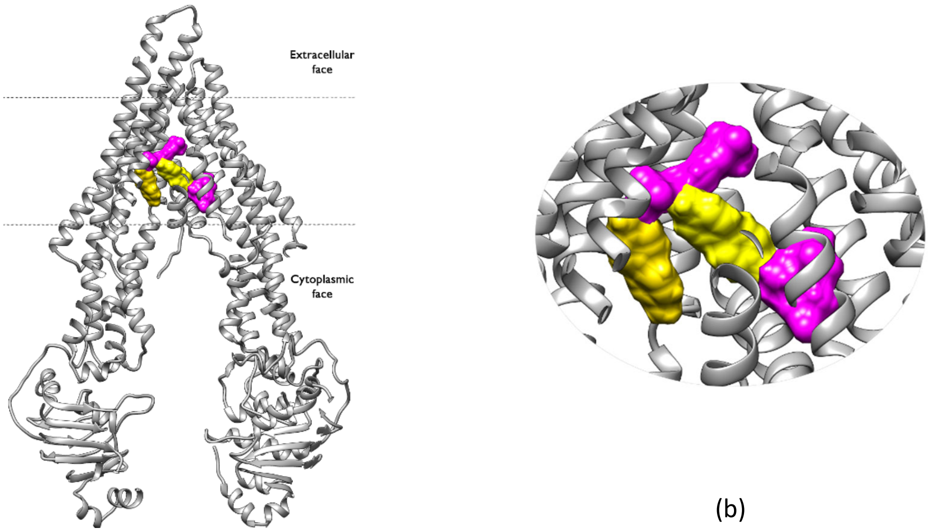

(a) (b)

Figure S4: BUS (magenta) and PQT (yellow) molecular surface representation of their different positions within the binding pocket during the 500 ns production run; (a) frontal view; (b) zoomed view; (c) view from the extracellular side of the protein looking into the inner chamber.

Compounds properties

Table S1: 2D structures of the molecules used in the study.

Name	Chemical structure
Amiodarone	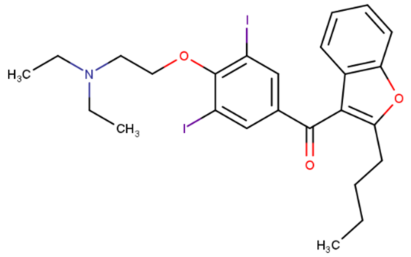
Carvedilol	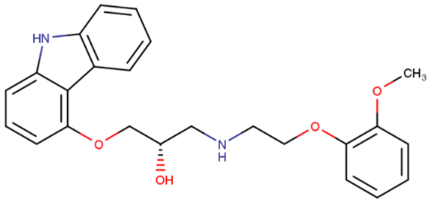
Cyclosporine A	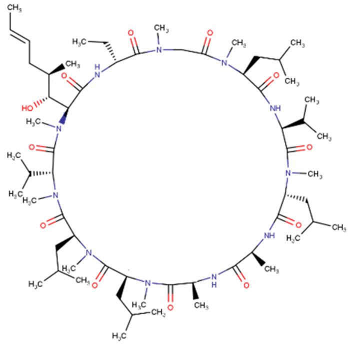
Doxorubicin	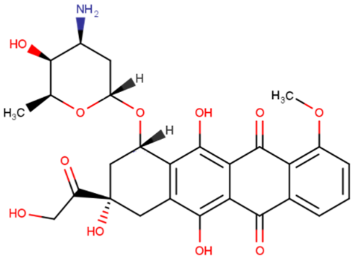
Pamidronate	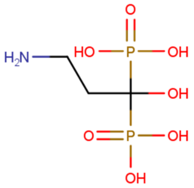
Busulfan	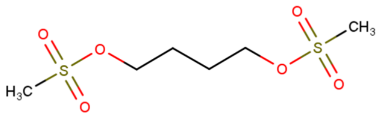

Gentamicin	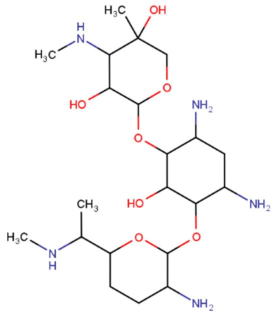
Paraquat	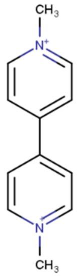
Valproic acid	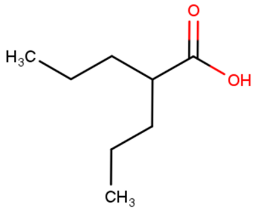

Table S2: Physicochemical properties of the studied molecules

	LogP	HBD[10]	HBA[11]	TPSA[12] (Å^2)	Heavy atom count	Aromatic rings
AMI[1]	7.57	0	4	42.7	31	3
CAR[2]	4.19	3	5	75.7	30	4
CSA[3]	2.92	5	12	279.0	85	0
DOX[4]	1.27	6	12	206.0	39	2
APD[5]	-4.70	6	8	161.0	13	0
BUS[6]	-0.52	0	6	104.0	14	0
GEN[7]	-3.10	8	12	200.0	33	0
PQT[8]	-4.22	0	0	7.8	14	2
VPA[9]	2.75	1	2	37.3	10	0

[1]Amiodarone; [2]carvedilol; [3]cyclosporine A; [4]doxorubicin; [5]pamidronate; [6]busulfan; [7]gentamicin; [8]paraquat; [9]valproic acid; [10]hydrogen bond donor count; [11]hydrogen bond acceptor count; [12]topological polar surface area.

MM/PBSA free energies of binding

Table S3: Free energies of binding and the various MM/PBSA terms. Estimate of the overall binding free energies for the ligand–P-gp complexes studied, using MM/PBSA calculations.

Name	ΔG_{Bind} (kcal/mol)	E_{VDW}	E_{elec}	$G_{non\text{-}polar}$	G_{Disper}	ΔG_{Gas}	ΔG_{Solv}	K_d calc (μM)	K_d exp (μM)
CSA[a]	-55.09	-105.28	-19.47	-80.32	149.98	-124.75	69.66	1.8×10^{-34}	0.2
AMI[b]	-31.23	-53.45	-10.36	-39.33	71.91	-63.81	32.58	2.9×10^{-17}	2.0
CAR[c]	-23.96	-41.38	-10.67	-32.24	60.33	-52.05	28.09	5.2×10^{-12}	0.3
DOX[d]	-24.79	-47.67	-9.85	-36.74	69.48	-57.53	32.74	1.3×10^{-12}	4.4
GEN[e]	-20.04	-41.02	-7.53	-35.14	63.65	-48.54	28.51	3.5×10^{-9}	-
BUS[f]	-12.77	-25.26	-3.29	-18.98	34.76	-28.55	15.78	6.1×10^{-4}	-
APD[g]	-15.44	-21.12	-9.62	-14.84	30.14	-30.74	15.30	7.2×10^{-6}	-
PQT[h]	-18.29	-20.03	-13.70	-15.09	30.54	-33.74	15.45	6.4×10^{-8}	-
VPA[i]	-9.15	-17.79	-2.51	-15.44	26.59	-20.30	11.15	0.24	-

[a]Cyclosporine A; [b]amiodarone; [c]carvedilol; [d]doxorubicin; [e]gentamicin; [f]busulfan; [g]pamidronate; [h]paraquat; [i]valproic acid.

NBDs distance

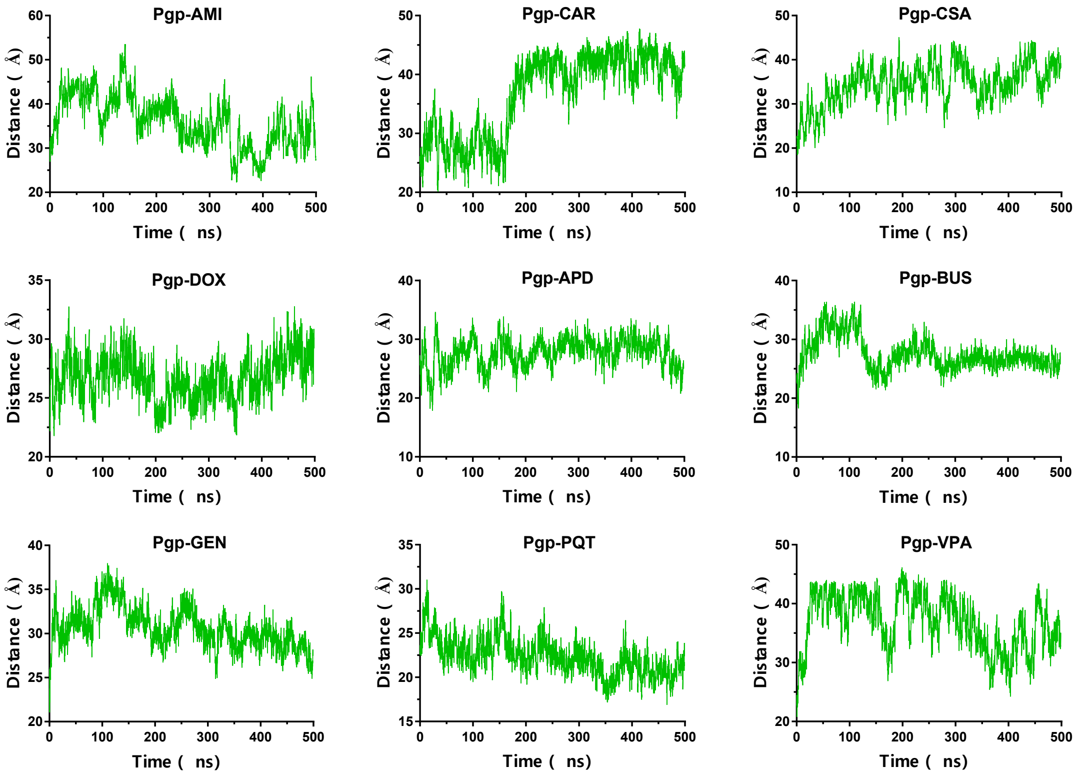

Figure S5: NBDs distance during the 500 ns production run. The separation was measured by the distance between the N atom in the Lys residue of the Walker A motif in NBD1 and the Cα of the

Ser residue in the signature motif of NBD2.

Principal Component Analysis

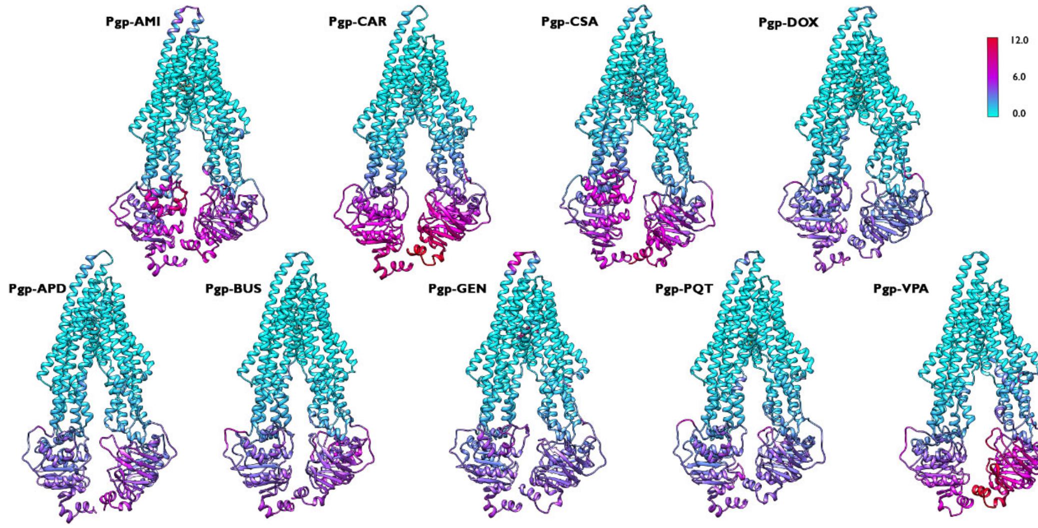

Figure S6: Cα-RMSF coloured representation for the P-gp–ligand systems along the principal components explaining at least 85% of the flexibility of the systems calculated from the 500 ns production run. The flexibility scale goes from cyan (lower values) to red (higher values).

Modes of Motion of P-glycoprotein

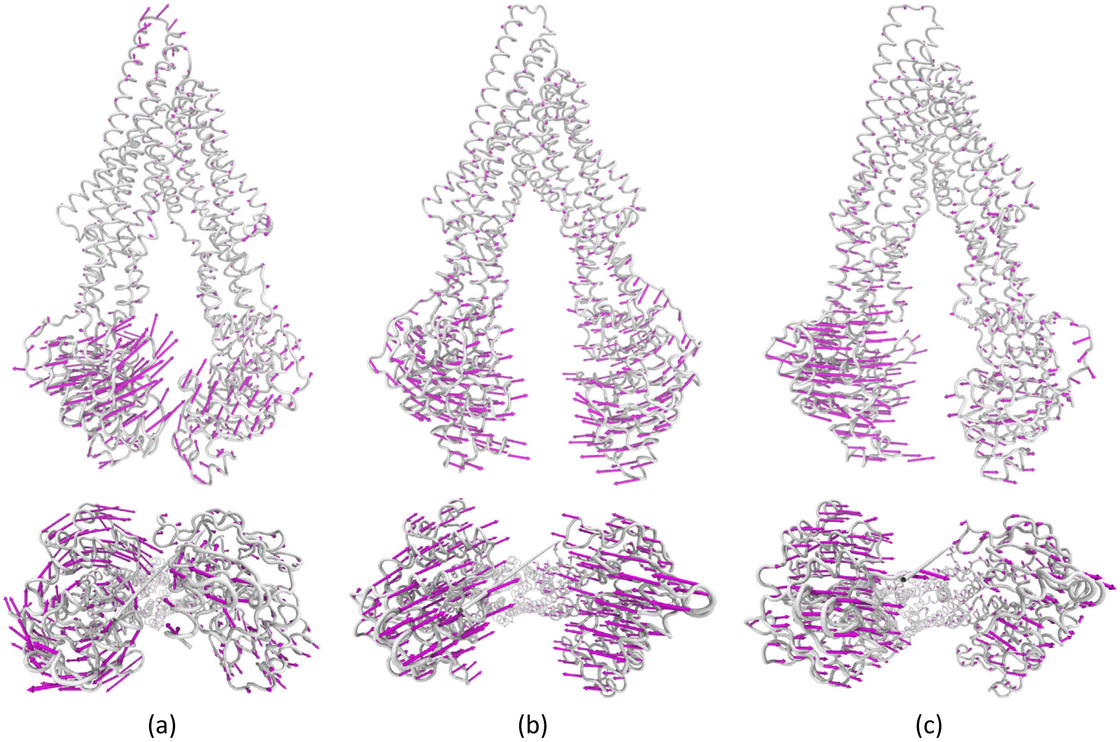

Figure S7: Front and Cytoplasmic view of P-gp motion patterns along PC1; (a) P-gp–AMI; (b) P-gp–CAR; (c) P-gp–CSA. The direction of the movement is represented by magenta arrows and the size of the arrows is proportional to the magnitude of the movement. For clarity, the reverse direction is not shown.

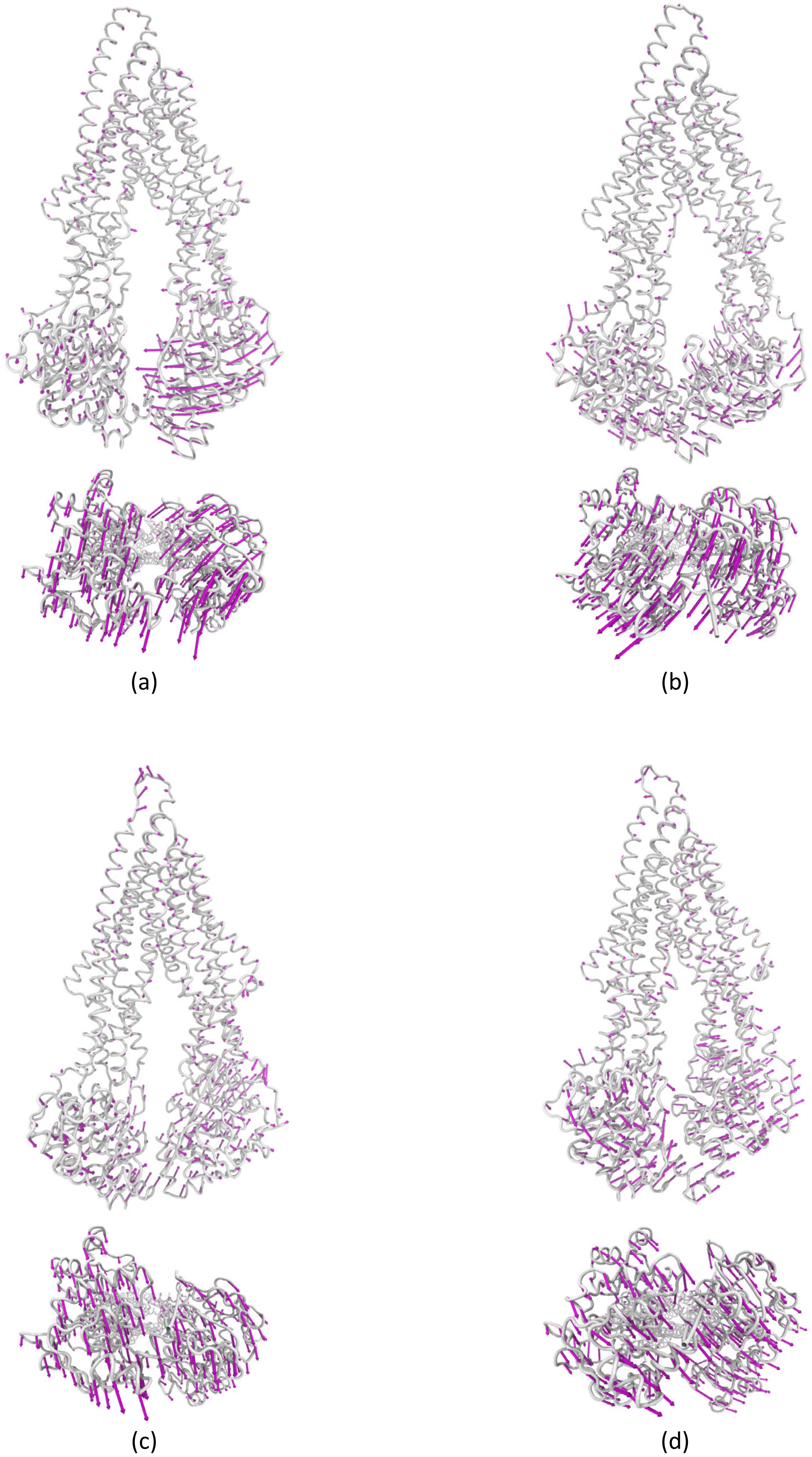
(a)
(b)
(c)
(d)

Figure S8: Front and Cytoplasmic view of P-gp motion patterns along PC1; (a) P-gp–APD; (b) P-gp–BUS; (c) P-gp–GEN; (d) P-gp–PQT. The direction of the movement is represented by magenta arrows and the size of the arrows is proportional to the magnitude of the movement. For clarity, the reverse direction is not shown.

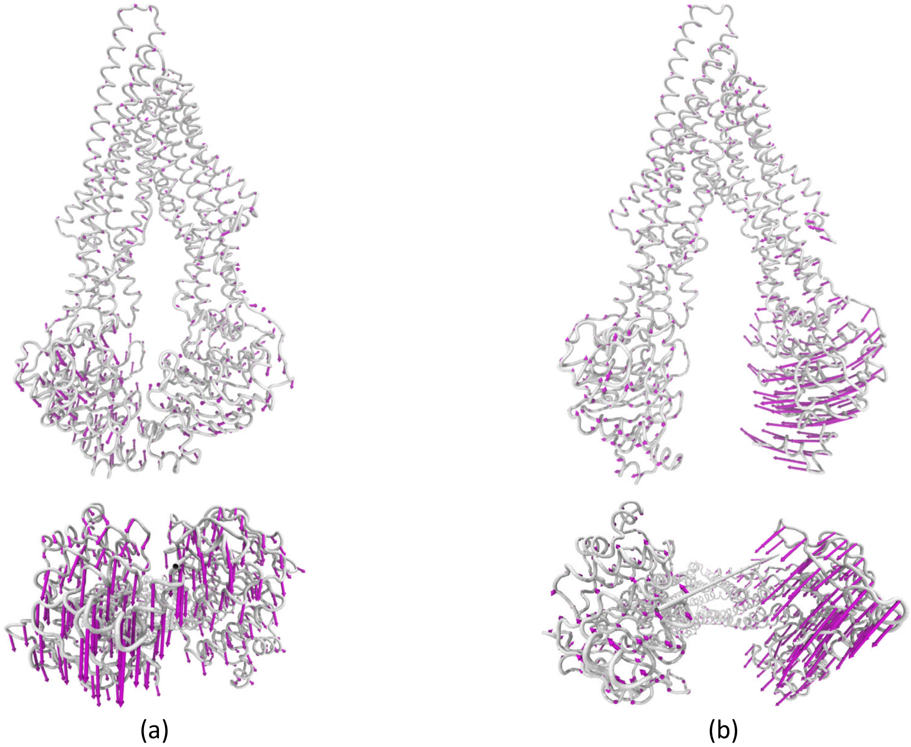

Figure S9: Front and Cytoplasmic view of P-gp motion patterns along PC1; (a) P-gp–DOX; (b) P-gp–VPA. The direction of the movement is represented by magenta arrows and the size of the arrows is proportional to the magnitude of the movement. For clarity, the reverse direction is not shown.

Binding Pocket

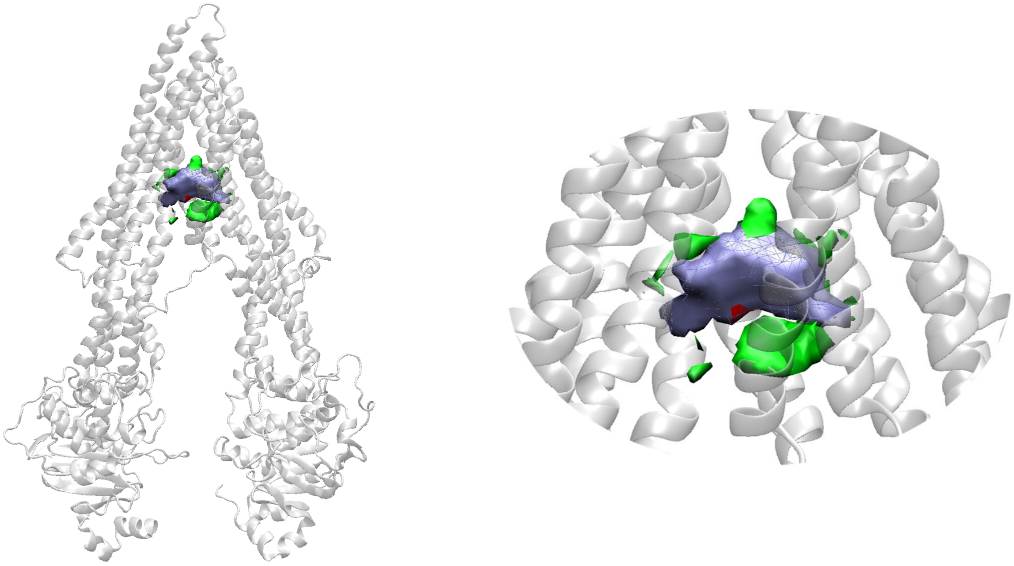

(a) (b)

Figure S102:Pocket shapes of the most-populated four cluster centroids for the active-bound systems; (a) front view; (b) zoomed view. The average pocket shape is shown as a blue surface. The additional regions that are more open or close than the average in each of the four clusters are shown as a green and red surface, respectively.

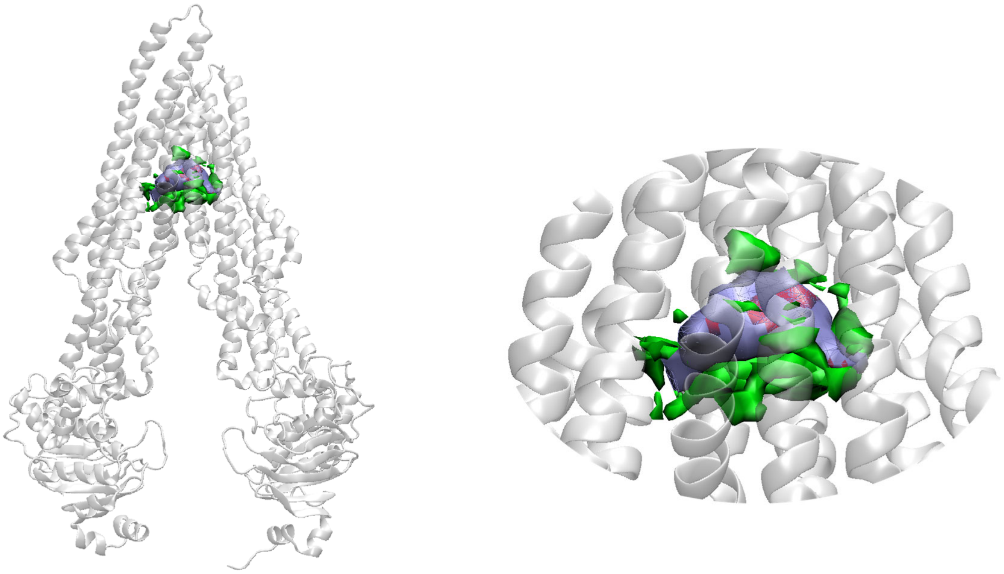

(a) (b)

Figure S11: Pocket shapes of the most-populated five cluster centroids for the non-active-bound systems; (a) front view; (b) zoomed view. The average pocket shape is shown as a blue surface. The additional regions that are more open or close than the average in each of the five clusters are shown as a green and red surface, respectively. The cluster centroids are much more open than the average shape compared to the active-bound systems, confirming the higher fluctuations of the non-active compounds within the internal cavity.

Solvent accessible surface area (SASA)

Table S4: Per residue SASA variations

Residue number	Average per residue SASA[12]								
	AMI[1]	CAR[2]	CSA[3]	DOX[4]	APD[5]	BUS[6]	GEN[7]	PQT[8]	VPA[9]
33	76.40	76.35	74.60	76.21	78.35	78.14	78.34	77.31	78.38
69	1.32	1.97	1.03	2.12	7.01	6.96	3.26	7.81	5.41
313	0.45	0.49	0.57	0.49	1.60	0.81	0.68	0.91	0.99
331	0.79	0.83	0.78	0.48	1.01	1.51	1.33	4.50	2.03
340	9.37	9.73	0.87	10.06	23.13	22.55	16.07	22.38	23.00
343	19.51	8.13	0.74	9.49	20.70	28.57	22.32	20.01	29.48
367	99.76	105.85	107.04	103.98	110.96	108.63	107.40	114.38	110.99
372	144.74	94.67	146.75	97.70	149.29	165.13	174.33	157.79	164.71
548	57.70	86.00	69.94	82.09	90.66	94.25	91.88	96.92	86.63
727	32.49	35.86	35.74	35.90	36.25	37.53	36.90	36.54	37.48
764	86.19	84.57	85.95	85.44	88.84	91.01	96.07	88.62	89.49
767	65.03	66.33	61.45	64.98	67.18	68.46	70.77	67.62	68.19
953	1.75	6.35	3.97	5.07	8.53	11.03	7.74	9.20	16.38
986	3.88	7.91	5.87	10.83	15.99	29.12	17.45	16.51	27.78

Residue number	Average per residue SASA[12]	
	Min(NA[10]) - Max(A[11])	% Decrease relative to Max(NA)
33	0.91	1.19
69	1.14	53.70
313	0.11	18.37
331	0.18	22.24
340	6.01	59.68
343	0.50	2.55
367	0.36	0.34
372	2.54	1.73
548	0.62	0.73
727	0.35	0.96
764	2.43	2.82
767	0.84	1.27
953	1.40	21.99
986	5.16	47.66

[1]P-gp–AMI; [2]P-gp–CAR; [3]P-gp–CSA; [4]P-gp–DOX; [5]P-gp–APD; [6]P-gp–BUS; [7]P-gp–GEN; [8]P-gp–PQT; [9]P-gp–VPA; [10]Non-active-bound system; [11]Active-bound system; [12] Solvent accessible surface area.

Table S5: Per residue SASA variations

Residue number	Average per residue SASA[12] AMI[1]	CAR[2]	CSA[3]	DOX[4]	APD[5]	BUS[6]	GEN[7]	PQT[8]	VPA[9]
38	47.78	46.18	48.30	47.63	42.49	45.82	45.53	46.00	35.60
184	39.37	49.12	67.59	40.81	36.45	25.02	34.34	30.22	32.35
188	38.64	35.27	39.26	36.05	32.10	29.39	21.38	26.46	31.23
320	8.44	7.69	8.26	8.29	7.51	6.21	5.24	5.32	4.81
391	49.94	39.60	40.22	40.36	38.24	31.79	37.17	36.95	36.40
420	30.49	38.18	32.18	33.64	10.85	5.52	29.27	7.32	28.27
559	62.70	95.91	73.74	71.91	44.18	10.17	5.78	49.84	39.52
580	45.61	42.93	56.46	39.21	23.57	13.20	22.59	18.83	23.87
581	2.58	0.44	1.16	0.40	0.07	0.10	0.20	0.13	0.25
601	4.97	5.84	6.96	6.29	1.69	4.89	2.92	4.58	2.28
1021	118.28	147.02	126.67	138.48	90.40	108.25	109.98	86.89	103.94
1040	0.12	0.09	0.12	0.10	0.01	0.06	0.07	0.02	0.05
1049	102.06	125.42	111.60	117.66	80.14	94.11	87.75	85.74	88.55
1090	124.00	123.14	124.24	124.84	110.54	121.46	122.47	118.19	121.07
1154	2.03	0.86	0.96	0.99	0.41	0.66	0.67	0.83	0.72

Residue number	Average per residue SASA[12] Min(A^k) - Max(NA^j)	% Decrease relative to Max(NA)
38	0.18	0.40
184	2.91	7.99
188	3.16	9.85
320	0.18	2.37
391	1.36	3.55
420	1.22	4.17
559	12.86	25.80
580	15.35	64.29
581	0.15	58.04
601	0.08	1.68
1021	8.30	7.55
1040	0.02	28.27
1049	7.95	8.45
1090	0.67	0.55
1154	0.03	3.53

[1]P-gp–AMI; [2]P-gp–CAR; [3]P-gp–CSA; [4]P-gp–DOX; [5]P-gp–APD; [6]P-gp–BUS; [7]P-gp–GEN; [8]P-gp–PQT; [9]P-gp–VPA; [10]Non-active-bound system; [11]Active-bound system; [12] Solvent accessible surface area.

Metrics of the clustering analysis

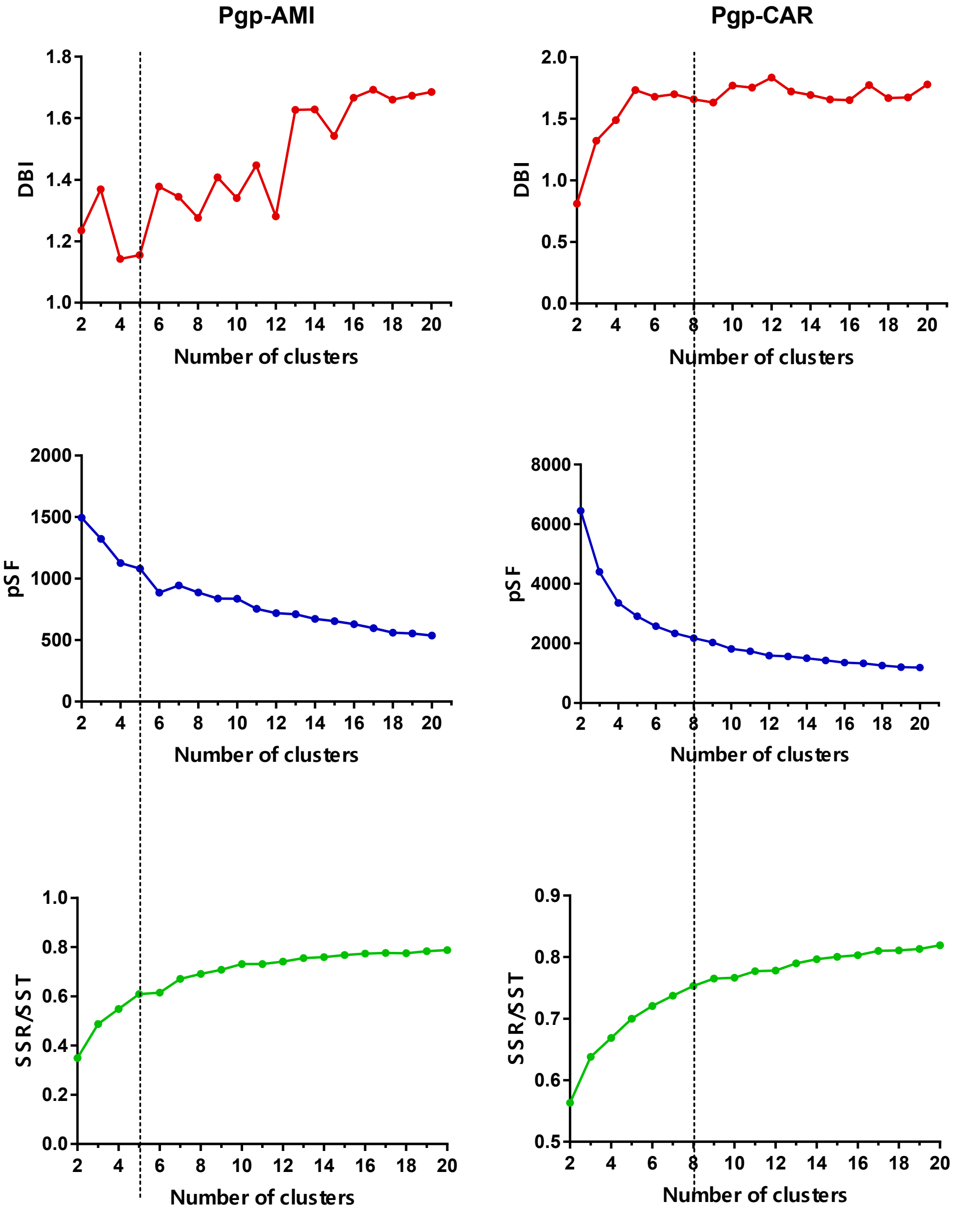

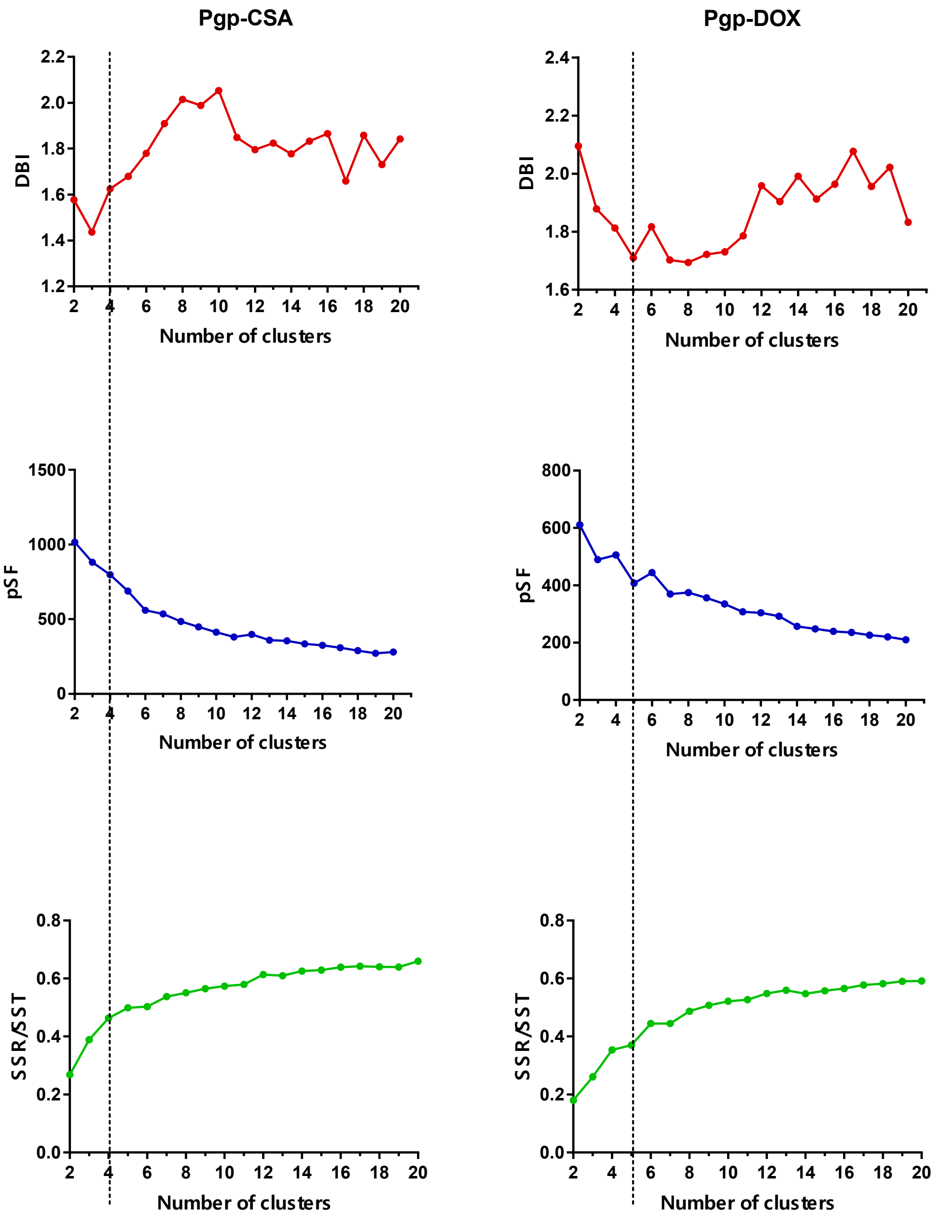
Pgp-CSA
Pgp-DOX
DBI
pSF
SSR/SST
Number of clusters

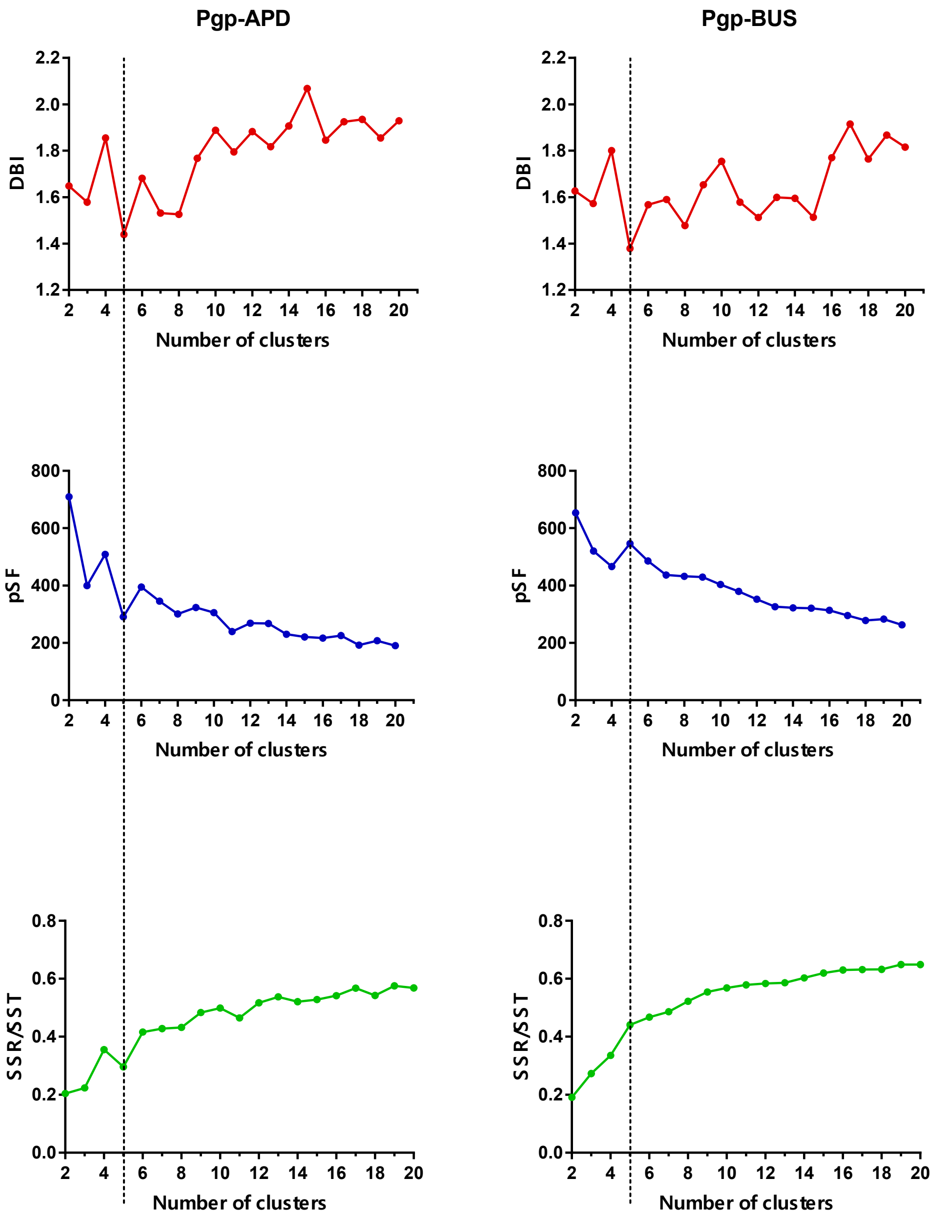

Pgp-APD
Pgp-BUS
DBI
pSF
SSR/SST
Number of clusters

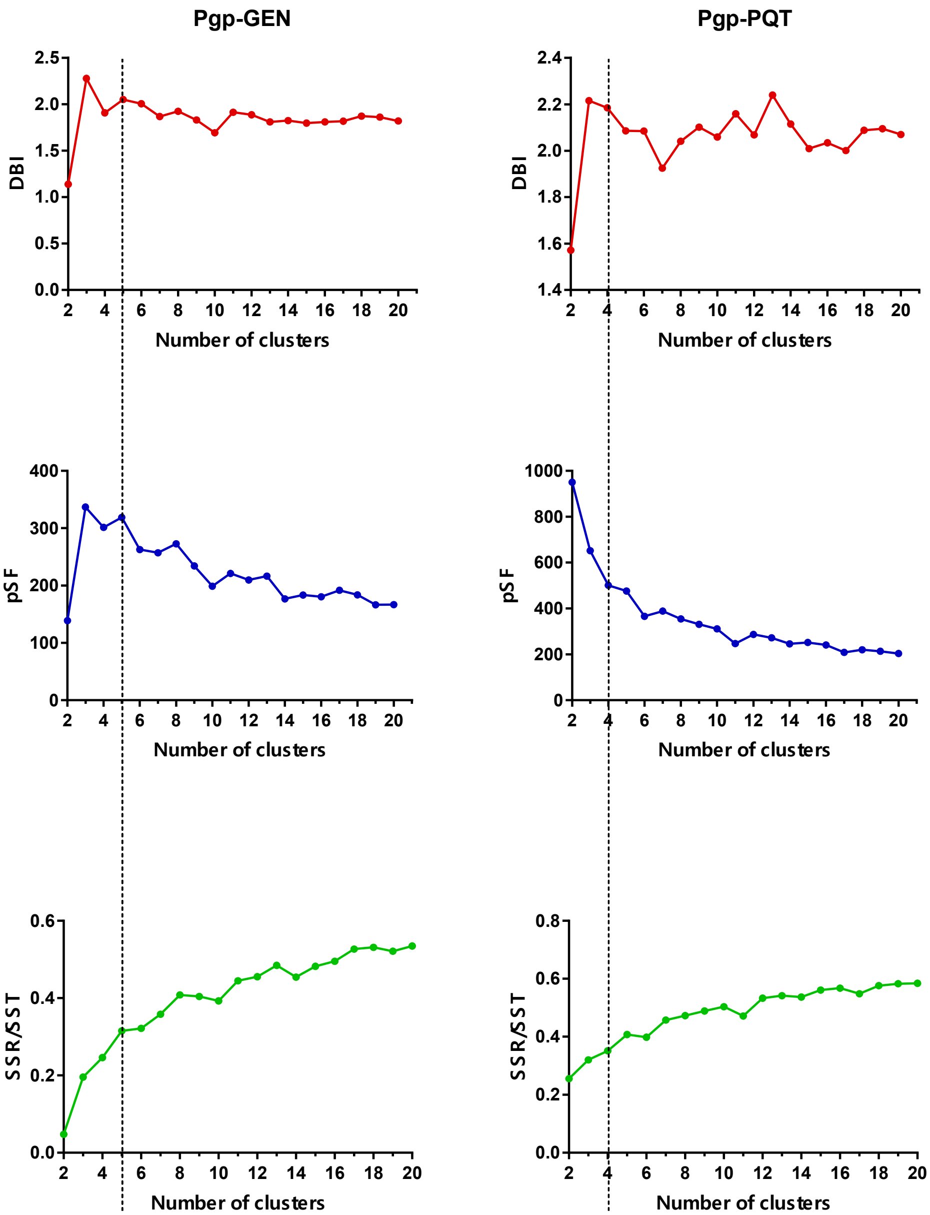

Pgp-GEN
Pgp-PQT
DBI
pSF
SSR/SST
Number of clusters

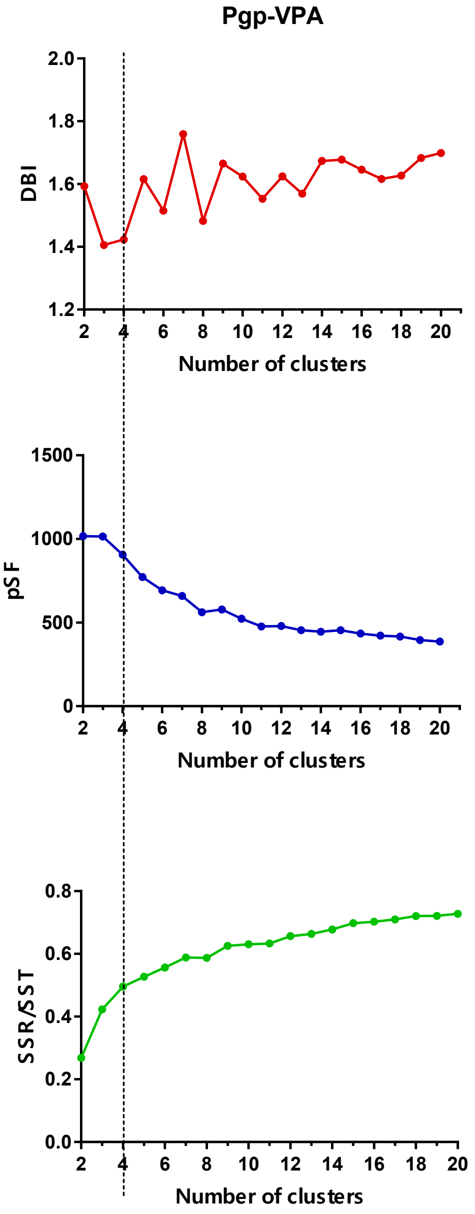

Figure S12: Metrics used to select of the optimal number of clusters of each ligand–P-gp system. The dashed lines in the graph indicate the selected number of clusters.
